# The Water Droplet Contact Line Probed with Multiwalled Carbon Nanotubes at the Air–Water Interface

**DOI:** 10.1021/acs.langmuir.5c03184

**Published:** 2025-10-04

**Authors:** Esa Hyyryläinen, Juha Merikoski, Markus Ahlskog

**Affiliations:** Department of Physics and Nanoscience Center, 4168 University of Jyväskylä, FI-40014 Jyväskylä, Finland

## Abstract

Within the issue of sessile droplet evaporation, particularly important is the behavior of the triple phase contact line governed by pinning phenomena. We demonstrate how pristine, insoluble multiwalled carbon nanotubes (MWNT) can exhibit ordering phenomena at the air–water interface, contribute to droplet pinning, and are also responsive to the evolving shape of evaporating water droplets at the contact line. The arc-discharge-synthesized MWNTs were of high quality, but they were mixed with graphitic impurity particles in the 10–100 nm size ranges. The MWNTs were ordered into chain structures by capillary interactions at the air–water interface. Moreover, we observed how the chain structures regularly turned perpendicular to the contact line a short time prior to the withdrawal of the strongly pinned contact line, which we explain with the capillary force acting in a region with nonconstant interface curvature. The MWNT chains thus offer a unique way to probe the local behavior of the droplet. We modeled the van der Waals interactions of MWNTs and graphitic impurities in the vicinity of the contact line. According to these, the related energies are large enough to explain issues related to the transfer onto the air–water interface. Capillary effects can be qualitatively explained by existing theories. As the MWNTs are strictly confined to the air–water interface, these results are complementary to the separate but closely related coffee ring effect.

## Graphical abstract

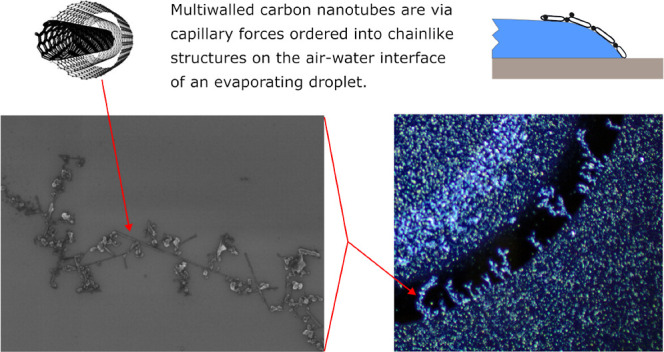

## 1 Introduction

The topic of sessile droplet evaporation is an old complex problem, which includes several quite distinct “subproblems”, such as the precise behavior of the triple phase contact line or briefly just the contact line. Since many of the subproblems have been possible to investigate thoroughly only with modern experimental methods and simulations, much progress is presently seen on this topic.

The mobility or dynamics of the contact line is often governed by pinning phenomena, caused by substrate heterogeneities.^1^ An evaporating droplet may shrink according to the constant contact radius (CCR) mode or constant contact angle mode, or some combination thereof.^2,3^ In the CCR mode, pinning dominates the contact line behavior. Pinning plays a key role in the well-known coffee ring effect, where colloidal nano- or microscale particles within an evaporating droplet of a base liquid form ring-like deposits.^4^ The deposits are formed when the withdrawing contact line is pinned for certain time periods. Substrate heterogeneities may influence pinning, but in the coffee ring effect, pinning is accentuated by the colloidal particles accumulating at the contact line.

Another distinct scenario could be considered for pinning of the contact line: A substrate with small (colloidal scale) mobile particles on the solid surface that the advancing contact line of a pure liquid droplet encounters and which then pins the contact line. Situations closely related to this scenario have been described in a number of articles, see, e.g., ref 5. However, in these works, the motivation has mostly been investigating how an advancing fluid interface on a substrate pushes on individual particles on it, mostly of larger size scales (>10 μm), whereby the relative influences of capillary, inertial, and other forces make the problem different than what is considered here.

In a few works,^6–8^ originally undertaken for purification purposes, we have demonstrated a system that seems to have the kind of effects that we are suggesting above. In these studies, we have on a hydrophilic substrate a uniform deposit of arc-discharge-produced multiwalled carbon nanotube (MWNT) material (Figure 1a,b), which contains high-quality MWNTs and also amorphous carbon particles (ACP). Henceforth, we use the phrase “MWNT material” for the mixture of MWNTs and ACPs, where the MWNTs are not easily purified or separated without damaging or contaminating them. When a water droplet is placed on any smooth substrate, the droplet at first expands to some maximum extension and later shrinks due to evaporation, and this of course happens with substrates carrying a MWNT material deposit as well. In this case, when the expansion rate has slowed down, most of the highly hydrophobic MWNT material is pushed by the advancing contact line and uplifted on the droplet surface, as shown in Figure 1c,d, and upon droplet drying, it is redeposited on the substrate. A most crucial observation is that from the original deposit to the redeposition, the MWNT material undergoes drastic changes in its internal state of aggregation (Figure S6 in the Supporting Information) but is at every phase two-dimensional in character, particularly at the air–water interface of the water droplet.

**Figure 1:**
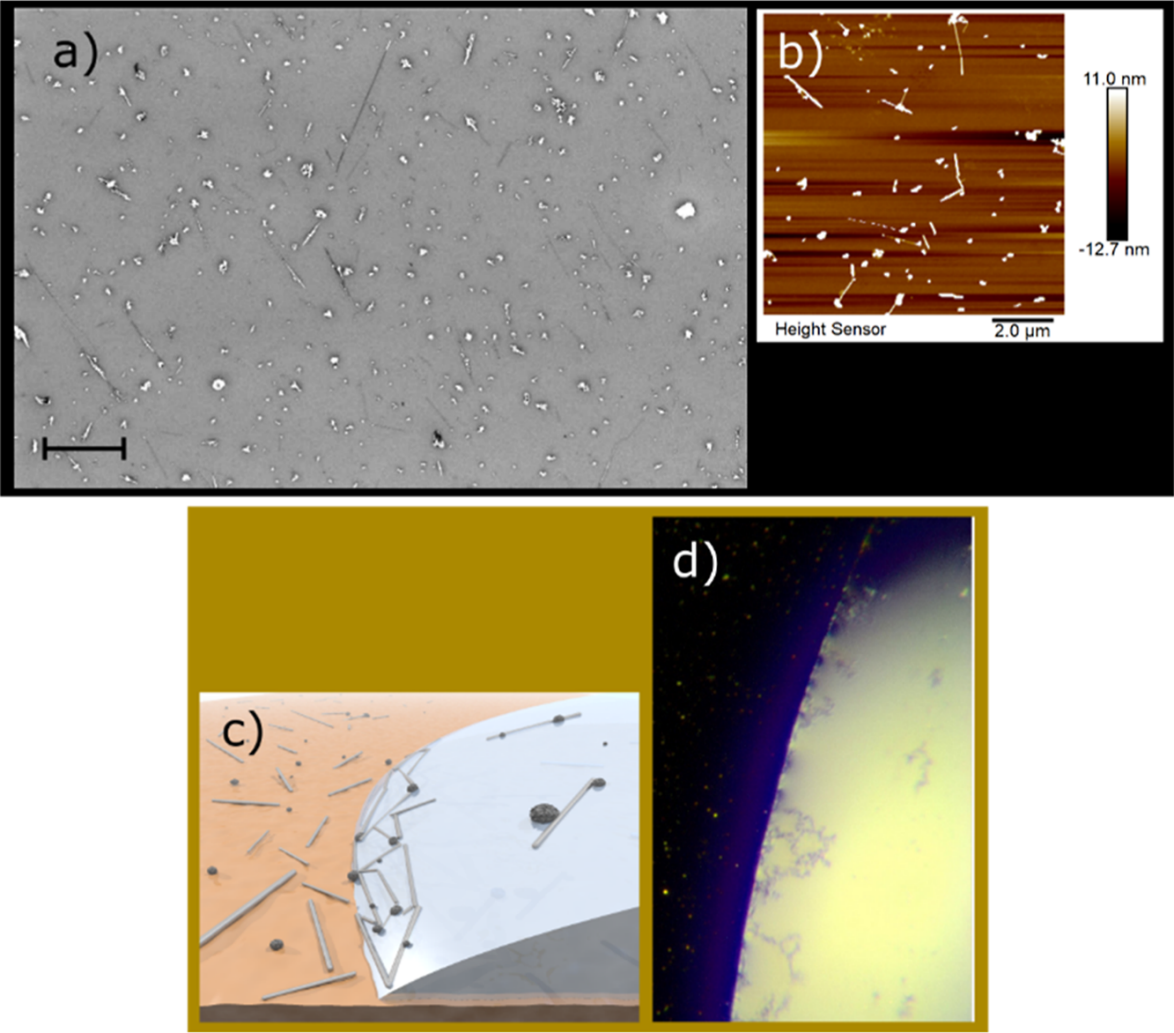
SEM ((a) scale bar 1 μm) and AFM (b) images of typical MWNT material deposits, with a spin-coated MWNT material on a Si chip. Besides MWNTs, there are plenty of separate graphitic ACPs. The figures below demonstrate what happens to these deposits in the first step of the experiments. (c) Schematic illustration of the expansion phase of the droplet, where the contact line advances over the MWNT deposit (not in scale). MWNTs and ACPs are uplifted from the substrate, and a few aggregates at the contact line but mostly are transferred higher up on the droplet surface. (d) The process depicted in (c) is demonstrated in a video still image via optical microscopy. Here, the reflection spot from the illumination source on the water interface crosses the field of view, giving an intense light from the surface of the water droplet perimeter region, where the MWNT material is clearly visible.

In our early work, we ignored the droplet behavior, as it was irrelevant to the original purification goal. In this work, this behavior, in particular upon evaporation, is at the focus of attention. We investigated the small part of the uplifted MWNT material that interacts with the droplet contact line. This MWNT material forms chainlike assemblies at the air–water interface due to capillary forces. The interplay of MWNTs and ACPs is found to be a key factor in their motion and ordering. We show that the related van der Waals energies are large enough to explain qualitatively the main experimental findings. We find that MWNT chains arise at the contact line shortly before the evaporating droplet begins to withdraw from its maximum extension and thus act as an indicator of the contact line behavior.

## 2 Materials and Methods

### 2.1 Experimental Section

We use the as-received arc-discharge-grown, commercially produced MWNT material that consists of both MWNTs and ACPs. It was dispersed via sonication into 1,2-dichloroethane with a concentration of 0.1 mg/mL. The MWNTs have a broad spectrum of lengths and diameters, but most of them are 0.5–3 μm long and 5–15 nm in diameter. The ACPs have sizes in the range 10 nm to 1 μm. Pieces of silicon wafer (“Si chip”) were prepared as substrates for deposition of the MWNT material, with precleaning and treatment with oxygen plasma to make them hydrophilic. The MWNT material was deposited on the chips by spin coating the MWNT dispersion in two rounds, which gave a suitable density of the deposits for this work. More details are given in Section Sa.

In the experiments, a droplet of ultrapure water was carefully placed from a pipet on the Si chips with MWNT deposits. The droplet volume was 3 μL, unless otherwise stated. As the droplet outward expansion slowed enough, the MWNT material was efficiently transferred onto the water droplet over the contact line. The experiment focused on the very minor part of the re-energized MWNT material that remained at the contact line. The droplet quickly reached a maximally steady form for a certain period and eventually dried away. A small section of the contact line region was followed from above with a long-range microscope equipped with a top-view camera. The experiments were performed in an atmosphere of controlled humidity, mainly at low (10%) and moderate (60%) relative humidity and room temperature.

After the experiment, the imprint on the MWNT deposit caused by the water droplet, including the MWNT material redeposited from the dried droplet, was imaged with optical microscopy in the dark-field mode, and fine details were imaged with scanning electron microscopy (SEM) or atomic force microscopy (AFM). The experimental data presented here represent a core set of about 30 samples that were investigated as described above. A much wider set of samples were studied less comprehensively (e.g., only with an optical microscope) as the experimental procedures were fine-tuned. More details are given in Section Sb.

### 2.2 Theory

We evaluate the van der Waals interactions for particles and interfaces present in the experiments. We use many formulas that can be found in ref 9 and reference to each equation in ref 9 used is given in Table S1 of Section Sf. Here, we give the equations in compact and more generic forms such that differences between the relevant geometries become obvious. The full interaction free energy *G* between two objects can be expressed as

1 $$G\left(l\right)=g_{k}\frac{1}{l^{z_{k}}}A_{k}\left(l\right)$$

where *g*_*k*_, *z*_*k*_ and $A_{k}\left(l\right)$ depend on the geometry *k* (flat, spherical, cylindrical) of the two objects involved and l is the distance between the objects. All dependence on the materials is in coefficient $A_{k}\left(l\right)$ . In the short-distance ( l→0 ) nonretarded limit $A_{k}\left(l\right)\to A$ , where the Hamaker constant *A* depends only on the materials. As magnetic properties are not important, one only needs the electric contribution^9^

2 $$A=\frac{3k_{B}T}{2}{\sum_{n}\sum_{q}\frac{1}{q^{3}}{\left(\frac{\varepsilon_{1}-\varepsilon_{m}}{\varepsilon_{1}+\varepsilon_{m}}\right)}^{q}\left(\frac{\varepsilon_{2}-\varepsilon_{m}}{\varepsilon_{2}+\varepsilon_{m}}\right)}^{q}$$

where in the sums *n* = 0, 1,...,∞ such that the first term in the sum is divided by 2 and *q* = 1, 2,...,∞ (often the term *q* = 1 gives the main contribution to *A*). In this formula, ε_1_ = ε_1_(*i*ξ_*n*_), ε_2_ = ε_2_(*i*ξ_*n*_), and ε_*m*_ = ε_*m*_(*i*ξ_*n*_) are the dielectric functions (of imaginary frequency) of the two materials 1 and 2 and the medium *m* (here air or water), respectively, and ξ_*n*_ are the Matsubara frequencies ξ_*n*_ = 4π^2^(*k*_B_ *T*/*h*)*n*. In the full retarded Lifshitz formulation with Derjaguin transform, we combine the most important formulas from ref 9 for object geometries *k* (flat, cylindrical, and spherical) at short distance l as

3 $$A_{k}\left(l\right)=\frac{3k_{B}T}{2}\sum_{n}{r_{n}}^{z_{k}}\sum_{q}\frac{1}{q^{3-z_{k}}}\int_{1}^{\infty }p^{z_{k}-1}\Delta_{1}^{q}\Delta_{2}^{q}e^{-r_{n}pq}\;dp,r_{n}=2l\sqrt{\varepsilon_{m}}\xi_{n}/c$$

where the factors Δ_*j*_ are (with materials *j* = 1, 2 and medium *m*, with *i* = 1, 2, *m*)­

4 $$\Delta_{j}=\left(s_{m}\varepsilon_{j}-s_{j}\varepsilon_{m}\right)/\left(s_{m}\varepsilon_{j}+s_{j}\varepsilon_{m}\right),s_{i}=\sqrt{p^{2}-1+\varepsilon_{i}/\varepsilon_{m}}$$

In Table 1, we give *z*_*k*_ and *g*_*k*_ for relevant geometries. The values of *z*_*k*_ and expressions of *g*_*k*_ for objects with finite radius ofcurvatures *R*_1_ and *R*_2_ follow from the Derjaguin transform. In the Derjaguin transform, two curved surfaces opposite to each other are replaced by small parallel (perpendicular to the distance of the objects) patches, and then the exact formula for two flat surfaces is used for the patches, resulting in a sum of interactions. The sum is then converted to an integral that can be done analytically by using an approximation valid for short distances $l\ll R_{j}$ ,^9^ resulting in eq 3. This condition for the distance is satisfied in the present work. The results of the Derjaguin transform are shown in Table 1.

**Table 1:** Purely Geometrical Parameters *z*_*k*_ and *g*_*k*_ for the Relevant Object Geometries^a^

geometry *k*	*z*_*k*_	*g*_*k*_	*g*_*k*_ for *R*_1_ = *R* and *R*_2_ → ∞
flat	2	*L* ^2^/12π	
cylindrical	3/2	$L{\left(R_{1}R_{2}/\left(R_{1}+R_{2}\right)\right)}^{1/2}\sqrt{2}/12\sqrt{\pi }$	$L\sqrt{R}\sqrt{2}/12\sqrt{\pi }$
spherical	1	(*R*_1_ *R*_2_/(*R*_1_ + *R*_2_))/6	*R*/6

a Here, *R*_1_ and *R*_2_ are the radiuses of curvature of object surfaces. For flat surfaces, *L* ^2^ is their area, and for cylindrical objects, *L* is the length of the object. The result for a flat surface is exact, and the others follow from the Derjaguin transform. The last column is for a long cylindrical and a spherical particle of radius *R* interacting with a flat surface (the radius of curvature of a flat surface is infinite).

At the relatively short distances l in our work, the difference between *A* of eq 2 and $A_{k}\left(l\right)$ of eq 3 is not very large (here up to 13%); for the actual values of *A* and $A_{k}\left(l\right)$ , see Table S2 of Section Sf. We prefer using $A_{k}\left(l\right)$ over *A* for better accuracy and to facilitate comparability with possible future work. For the dielectric function ε_*j*_(*i*ξ) of each material *j*, we have used the parameterizations given in ref 10. Tests of our methods, alternative approaches, and technical questions are discussed in Section Sf. The numerical parameters related to each geometry are as follows. The length of an MWNT is *L*_C_ = 1 μm, its radius is *R*_C_ = 5 nm, and the radius of an ACP is *R*_S_ = 20 nm. The distance of the nanocarbon objects from the substrate is $l_{S}$ = 0.6646 nm and from water $l_{W}$ = 0.32 nm, values deduced from experiment and simulation, respectively (see Section Sf). The difference between these distances favors the contact with water, as computed from the direct l dependence $l^{-z_{k}}$ in eq 1 for cylindrical and spherical geometry, namely, for an MWNT ${\left(l_{W}/l_{S}\right)}^{-3/2}\approx 3.0$ and for an ACP ${\left(l_{W}/l_{S}\right)}^{-2}\approx 4.3$ .

## 3 Results

We present the experimental results as follows: (Section 3.1) an outline of the droplet evolution, whereby we also describe how the bulk part of the uplifted MWNT material distributes on the droplet during the different phases, (Section 3.2) a description of the MWNT chains, and (Section 3.3) their interaction with the contact line at specific moments within the droplet evaporation.

### 3.1 Phases of the Evaporating Droplet

Figure 2 shows schematically the main phases of the experiment, those of the expansion, standing, and withdrawal phases of the droplet, that follow when a water droplet is carefully placed on the chip with the MWNT deposit and allowed to evaporate. The scheme also depicts the behavior of the bulk part of the displaced MWNT material, but for clarity, the relatively small MWNT chains are not yet shown in this figure.

**Figure 2:**
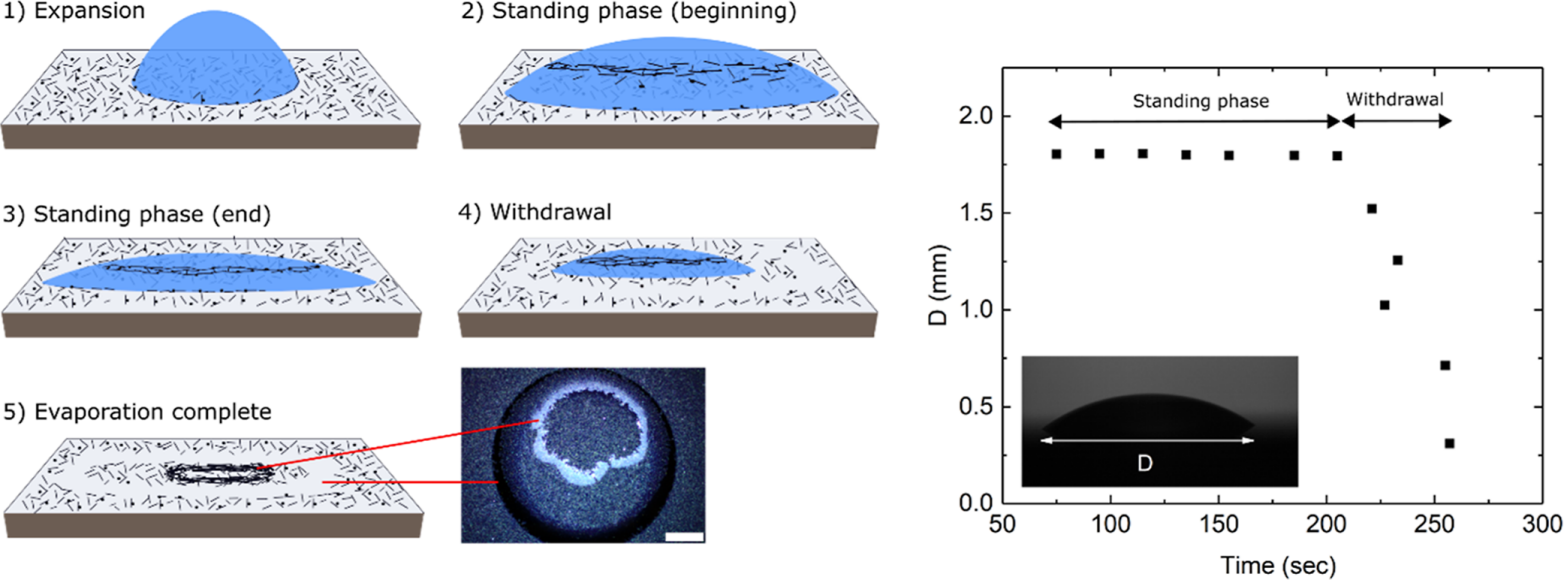
Left: Schematic illustration of the main phases (1–4) of the experiment and the resulting imprint (5) on the MWNT deposit. A water droplet is placed on a MWNT deposition, whereby the MWNT material (sticks for MWNTs and dots for ACPs) is accumulated on the droplet surface and later redeposited on the substrate. The MWNT chains have for clarity been left out of the figures. Next to schematic subfigure (5) is an optical image of a sample after the experiment (scale bar 0.5 mm). The red lines connect the annulus and the main part of the redeposited MWNT material to the corresponding parts in (5). Right: Qualitatively typical data for the diameter of a droplet on a deposit of the MWNT material as a function of time. This droplet was atypically small since accurate data was then easier to measure with our setup. The fast expansion phase is not shown here.

**(1) Expansion phase**: Initially, the droplet spreads in a few tens of milliseconds into a shape with a circular contact line, whereby the MWNT deposit under the forming droplet is mostly unaffected. The expansion then continues much slower for a period of ≈30 s. We have described this phase in good detail in ref 6 and present some complementary data also in the Supporting Information. At this point, the advancing contact line interacts with the hydrophobic MWNT material on the hydrophilic substrate, whereby most of it transfers (is lifted) onto the droplet surface, as described in refs 7 and 8 . The area that is thus emptied results in a ringlike pattern, an annulus, which is a dominating feature in the final imprint on the MWNT deposit.

**(2–3) Standing phase**: This begins when the contact line of the droplet attains a maximal diameter. The pinning of the contact line is undoubtedly affected by the accumulation of the material at the contact line from the MWNT deposit. However, most of the uplifted material from the expansion phase has moved away from the droplet perimeter and contact line and forms (typically) an irregular circular raft higher up on the droplet surface. The droplet evaporates at a constant (in plane) radius while the height decreases, at a rate which of course depends on the humidity and other factors, but always this takes around 80% of the time of the entire process. The droplet perimeter becomes unstable when the contact angle is reduced to some critical value. Thus, the samples strongly exhibit a CCR-type behavior.

**(4–5) Withdrawal phase**: This begins as the contact line starts to recede. In Figure 2, we also show typical data for the droplet diameter as a function of time. The MWNT material raft on top of the droplet is redeposited on the Si surface as the withdrawal proceeds, and the droplet eventually dries up. One example of this redeposition is seen in the optical micrograph of Figure 2 as the bright-colored, often ring-shaped overlay. These redepositions are of little interest in this work, but they are for completeness discussed in Section Sc.

We have shown in a previous work^6,8^ that the outer diameter of the annulus corresponds to the maximum extension of the droplet. The contact angle, just a few degrees on the freshly prepared hydrophilic silicon substrate, is quite steadily around 20° when the hydrophobic MWNT material has been deposited. We estimate the dimensions for a 3 μL sessile droplet, in the beginning of the standing phase, as follows: the diameter, as measured from the annulus outer diameter, was 4 ± 0.5 mm (Figure S2), and the height, as estimated from calculated values, assuming a spherical cap and the typical contact angle, was around 0.4 mm. The annulus width varied within 100–500 μm (for 3 μL droplet).

### 3.2 MWNT Chains

While, as stated above, the overwhelming majority of the uplifted MWNT material diffuses toward the upper parts of the droplet, there is visibly some aggregation at the contact line. We show in the next section (Section 3.3) that MWNT chains form into their final shape from this residual aggregation. Here, we present their structure.

The chain structures, shown in Figure 3a–c, are typically solitary, often branched, and perpendicular to the perimeter line. The length distribution spans a range of ca. 10 μm to more than 200 μm. The MWNT material deposition in its original form is shown in Figure 1. Here, the ACPs are separate from the MWNTs, while in the higher resolution SEM images of Figure 3, we can observe that practically all ACPs have been attached to the MWNTs, which is a general feature. When it is appropriate to emphasize this, we may thus speak, instead of MWNTs, of MWNT/ACP complexes, which have a couple of ACPs of size 10–100 nm randomly placed along the MWNT. The clear visibility in the optical microscope of the MWNT chains is mainly due to the scattered light from these ACPs.

**Figure 3:**
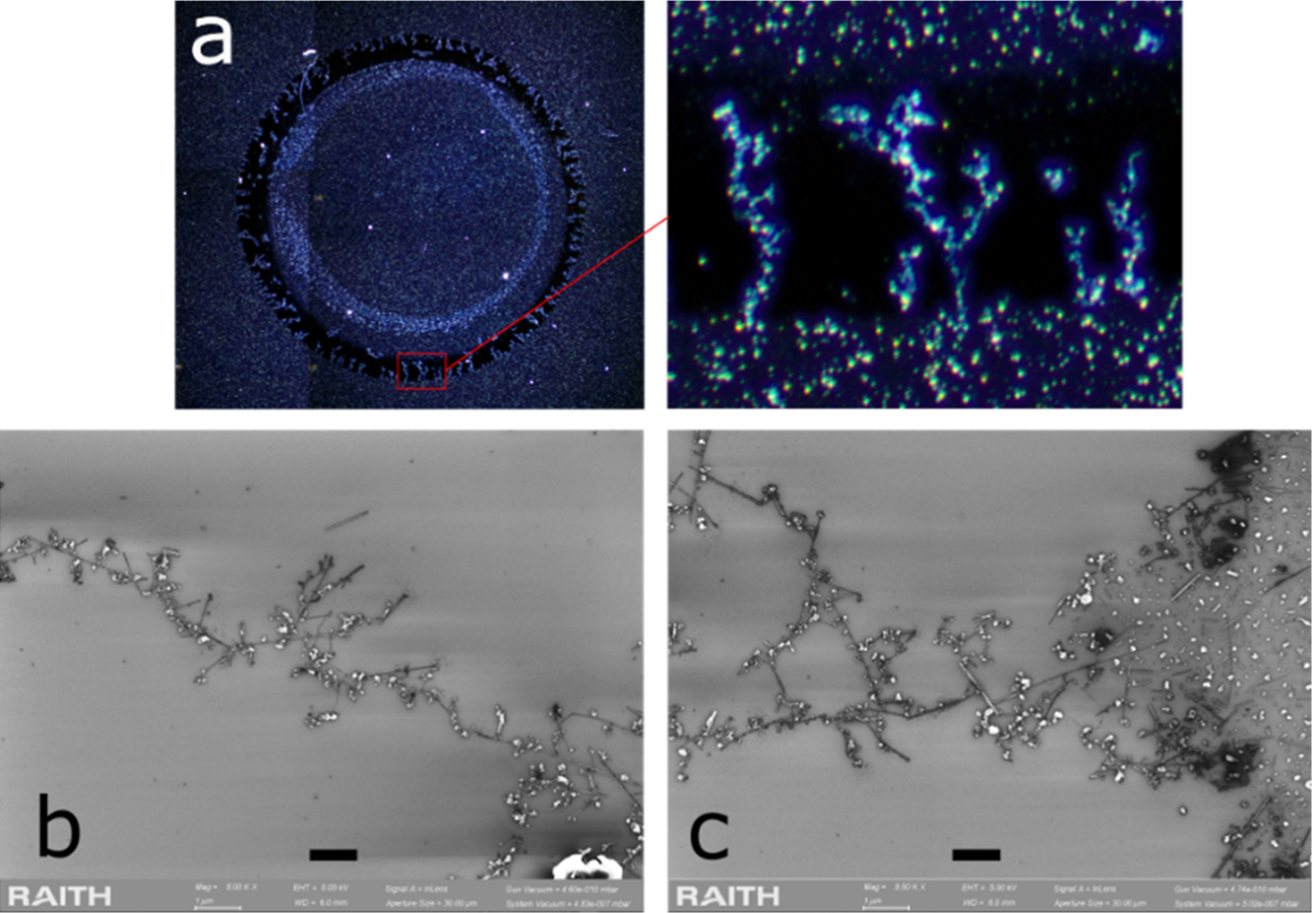
(a) Optical micrograph of an example of an imprint in a MWNT deposit after the experiment. The close-up view of the rectangular section in the lower part of the annulus shows in detail the MWNT chains pointing inward. (b) SEM image of part of a typical MWNT chain structure. (c) Image from the section where the chain begins from the outer perimeter of the annulus. Scale bar 1 μm.

From the morphology of the MWNT chains, one can infer that an apparent end-to-end attraction between the individual MWNTs (that is mostly MWNT/ACP complexes) has been a driving force during their formation. There is also a tendency of single MWNTs to occasionally place themselves roughly perpendicular to the main chain. Another feature is the branching of the chains, leading to network formation. The structure is irregular, especially with the randomly placed ACPs, but one can firmly conclude that the MWNT chains and the strands of the networks are effectively one-dimensional, consisting of roughly end-to-end connected MWNTs. More imaging data on the MWNT/ACP complexes and MWNT chains is presented in Section Se.

We demonstrate in the Supporting Information that the MWNT chain structures appear regularly at the droplet perimeter only in samples with low-density deposits of the MWNT material. The sample in Figure 2 is a higher density sample (which can be inferred from the very dense and bright redeposit), and consequently, there are very few chain structures in the annulus. On the other hand, in the low-density sample in Figure 3a, the MWNT chains heavily populate the annulus.

### 3.3 Dynamics Prior to Droplet Withdrawal

This section contains our central experimental results. We show how the MWNT chain structures form in a manner that follows the evaporating droplet shape. Figure 4 shows video still images in the region of the contact line from different moments in the second half of the droplet standing phase (the video sequence is available in the Supporting Information). Figure 4a is at a moment roughly in the middle of it. Mobile fragments of the MWNT material are still close to the contact line, while the aggregation of the MWNT material at the contact line is also visible. Figure 4b,c is taken from moments quite close to the end of the standing phase and the withdrawal of the droplet. The MWNT chains emerge on the droplet surface from the aggregations at the perimeter, although how exactly they are formed, prior to their straightening, remains a mystery with the present optical microscopy techniques we use. One end of the chain remains fixed to the contact line, while the rest of it turns to point roughly perpendicularly inward. This straightening procedure occurs within the last quarter of the standing phase time.

**Figure 4:**
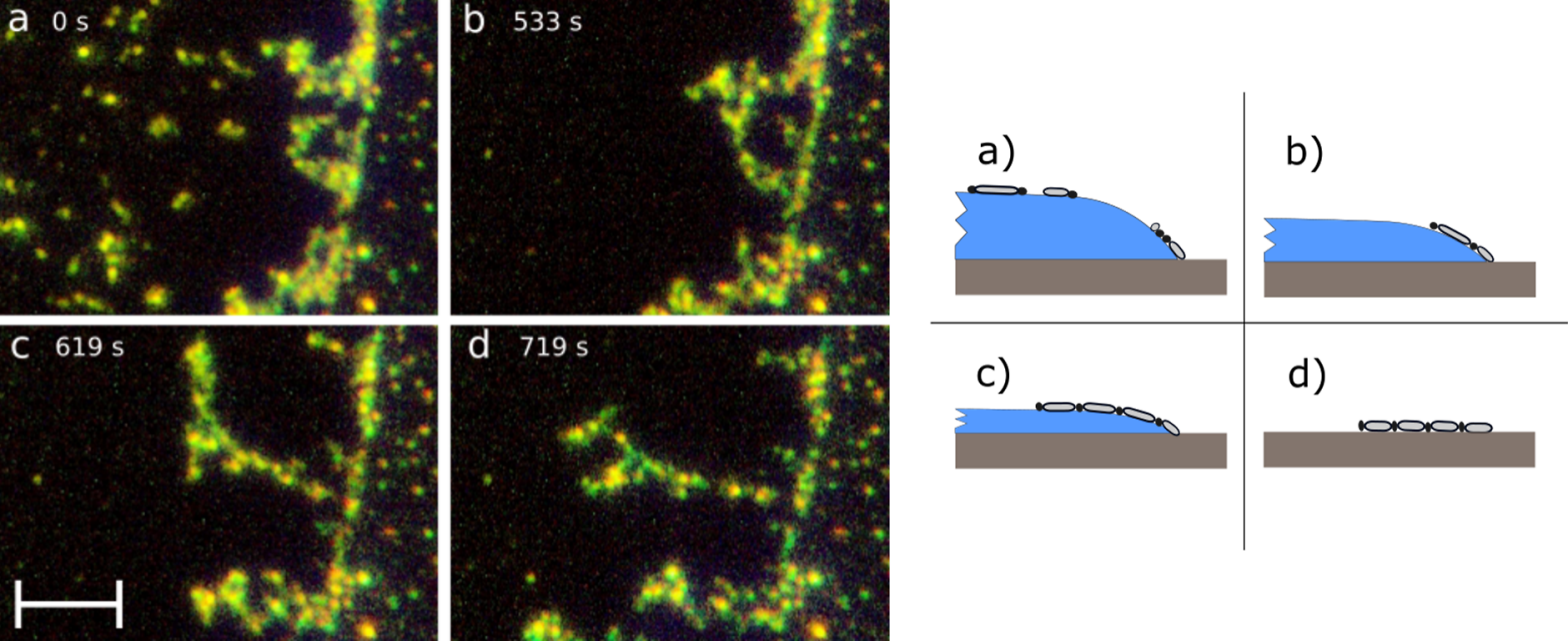
Left: Video still images via optical microscopy of MWNT chain formation at the edge of a water droplet during the standing phase (a–c) and after drying (d). Schematic figures (not in scale) on the right depict the corresponding state of evaporation of the droplets and the MWNT chain at the droplet edge. The time (begun in the first image) is indicated in the upper left corner. The contact line goes along the bright vertical traced line, slightly tilted to the right. At stage (a), some MWNT material has aggregated to the contact line, while separate fragments can still be seen higher up on the water surface. At later stages (b,c) toward the end of the standing phase, the MWNT chains straighten up. Figure (d) depicts the redeposited chain on the substrate immediately after the droplet has withdrawn. The chain is slightly sharper in appearance as it is now well in the focus of the microscope. The scale bar in the optical image is 50 μm.

The sequence of Figure 4a–d shows clearly how the MWNT chains react with the droplet evolution at the contact line, but here the water interface is rather invisible. Figure 1d is very helpful in this matter. Normally, the illuminating light beam is positioned on the water interface so that the main reflection spot is away from the images. In Figure 1d, the reflection spot crosses over the field of view while simultaneously illuminating the water interface and the MWNT chain structures as well.

Quite immediately after the chains have straightened out, the contact line of the thinned-out droplet edge can be observed to withdraw, and the chains are redeposited on the substrate. In Figure 4d, the droplet has entirely withdrawn, and the seemingly intact MWNT chain lies on the substrate, identical to those of Figure 3a. The chain formations thus survive as they are redeposited on the substrate.

On the water surface, the MWNT chains can be imaged only optically, with limited resolution and a little out of focus due to the droplet curvature. The higher resolution SEM images are available only after the redeposition process, and hence, the exact relation of the redeposited chains to their prior morphology on the water droplet surface is somewhat uncertain. Nevertheless, the chain and network patterns, as seen in the optical microscope and SEM images, are consistently the same, whereby it is clear that the redeposition onto the substrate process cannot strongly shift the MWNTs.

The data we have shown so far demonstrate the ordering processes and movement of the MWNT material but cannot, at least directly, tell what possible role the flow of the liquid has, which generally is present in an evaporating droplet. In Section Sd, we show evidence for flow in the radially outward direction well below the surface. It is expected as a general feature in evaporating droplets with a contact angle of a wetting liquid, where the outward flow sustains an evaporative flux that has a maximum at the perimeter.

The data demonstrate quite typical CCR-mode behavior for water droplets that are strongly pinned to the maximal perimeter. The MWNT material aggregation at the perimeter is an obvious contributor to this pinning, and, moreover, the MWNT chain alignment at the perimeter of the droplet during the prewithdrawal stage (Figure 4) is to our knowledge a new type of dynamic indicator of the droplet evaporation mechanism that builds on the interaction between the MWNTs and the water surface with a changing curvature.

We aim to demonstrate below that the transfer of the MWNT material to the droplet surface (Section 3.4) and its role in the droplet pinning mechanism (Section 3.5) are feasible, in agreement with computed van der Waals interactions. Moreover, we demonstrate the role of the capillary force in directing the MWNT chain behavior upon droplet evaporation (Section 3.6). The MWNT chains themselves will require separate treatment, but we consider here also a rudimentary explanation of them (Section 3.7). Finally, we briefly compare our results with the relatively established topic of the coffee-ring effect (Section 3.8).

### 3.4 MWNT Material Transfer onto the Water Surface

Nearly all of the MWNT material is lifted onto the droplet surface at the slower phase of the expansion phase (Figure 2). Moreover, once at the air–water interface, practically all the ACPs merge with the MWNTs to form MWNT/ACP complexes, as was described in Section 3.2.

These observations can be explained by calculations of the various interactions in the relevant geometries. We computed theoretical estimates of the van der Waals interactions between objects of interest using the full retarded Lifshitz theory with retardation^9^ as described in Section 2.2 and further elaborated in Section Sf. Here, we concentrate on short-distance interactions for MWNTs and ACPs on the surfaces of the substrate and the droplet as computed from eqs 1, 3, and 4 with geometric factors in Table 1. Our main results for the order of magnitude of attractive short-distance interactions are as follows (we discuss the details of the calculations with critique in Section Sf):

1. For a typical MWNT (length 1 μm, radius 5 nm) on the substrate, the free energy of attraction is *G*_ms_ ∼ 100 eV. For an MWNT on the water surface, we find *G*_mw_ ∼ 200 eV. For an MWNT inside water on the substrate (under the droplet), *G*_mi_ ∼ 20 eV.
2. For a typical ACP (radius 20 nm) on the substrate, we find *G*_as_ ∼ 1 eV. For an ACP on the water surface, *G*_aw_ ∼ 2 eV. For an ACP inside water on the substrate (under the droplet), *G*_ai_ ∼ 0.2 eV.
3. For an ACP attached to an MWNT, we get *G*_am_ ∼ 2 eV in vacuum and *G*_amw_ ∼ 1 eV inside water. Note, however, that the irregular shape of ACPs (compared with a spherical shape used in the model) can in some cases increase the interaction energies (also in item 2 above) as discussed in Section Sf.

These values are estimates of energies that can be compared with *k*_B_ *T* at room temperature and used for establishing their relative order. Item 1 shows that the MWNTs are, due to their length, strongly bound to the substrate and to the water surface, while the interaction between an MWNT and the substrate inside water (under the droplet) is much weaker. We conclude that the estimates given above are in harmony with the process of MWNT transfer to the water surface.

The starkly hydrophobic MWNTs and ACPs residing on the air–water interface should develop capillary interactions between them, apparently leading to the MWNT/ACP complexes mentioned above. Long-distance interactions also depend on the particle geometry, which is widely different for MWNTs and ACPs, cf. items 1 and 2 above for short-distance interaction energies. We estimate here that the free energy of the van der Waals attraction for an ACP attached to an MWNT is ∼2 eV in vacuum and ∼1 eV inside water (item 3), so we conclude that an ACP once driven to contact with an MWNT stays in contact with it.

The order of magnitude of the van der Waals energies discussed above varies from 1 to 200 eV. In addition to them, there are other interactions. The short-range hydrophobic attraction^11^ that is not included in our description would increase the total energy of attraction in the case of a water surface. The particles slightly deform the water surface, which would further increase the attraction and produce capillary forces between the particles. The effect of the line tension is expected to be relatively small.

### 3.5 Droplet Shape and Pinning

The common view is that the profile of the droplet closely follows that of a spherical cap (unless the withdrawal of the contact line is fast). In the CCR mode, the contact angle concomitant with this geometry decreases steadily.^2,3^ For a spherical cap, the principal radii of curvature are the same, *R*_1_ = *R*_2_ (see Figure 5a). We shall denote the principal curvatures by ${c_{k}=1/R}_{k}$ . As discussed later, during the various phases of the drying process in our experiments, the values of *c*_1_ and *c*_2_ can locally deviate considerably from those of the spherical cap model.

**Figure 5:**
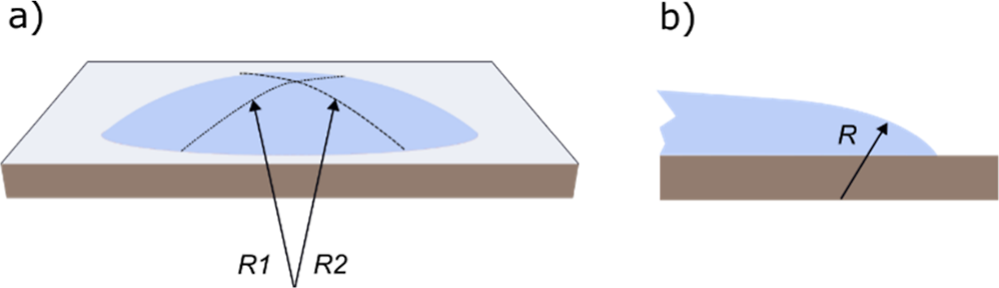
(a) Schematic image of the principal radiuses in an ideal droplet following a spherical cap geometry, where *R*_1_ = *R*_2_ at each point of the surface. (b) Cross-sectional view of a local deviation of the curvature at the perimeter of an evaporating droplet, with a significantly reduced radius of curvature.

It is known within the topic of capillary interactions that local changes in interface curvature tend to drive particles toward regions of larger mean curvature *H* and deviatoric curvature Δ*c*,^12^ defined as

5 $$\Delta c=c_{1}-c_{2}=\frac{1}{R_{1}}-\frac{1}{R_{2}}$$

For the spherical cap model, *c*_1_ = *c*_2_ and Δ*c* = 0, but even under ideal experimental conditions, the values of the mean curvature *H* = (*c*_1_ + *c*_2_)/2 and in particular Δ*c* deviate from those for the ideal spherical shape, especially close to the droplet perimeter, see Figure 5b. First, at nanoscale and close to the contact line, the shape of the interface is expected, due to effects often related to line tension, to deviate from that of a spherical cap, leading to nonzero Δ*c*. In addition, as shown in Section Sg, the typical value of the Bond number in our experiments, Bo = 4.5, is large enough to lead to larger *H* and Δ*c* close to the contact line.

Even larger value of Δ*c* results locally from the presence of MWNT material aggregation at the perimeter of the droplet (see Section 3.3) as the contact line gets locally pinned by the particles on the substrate (see Section Sh). The local deviations in the droplet shape and Δ*c* can mostly not be observed directly. However, as Figure 6 shows, the resulting deviations in the shape of the perimeter line in the imprint on the MWNT material deposition are well observable. In Figure 6a, the deviations have been particularly strong, and they are therefore well visible in the optical micrographs. The SEM image in Figure 6b shows that at a smaller scale, the deviations are omnipresent and often associated with MWNT chains. One can see a similar deviation in Figure 3c.

**Figure 6:**
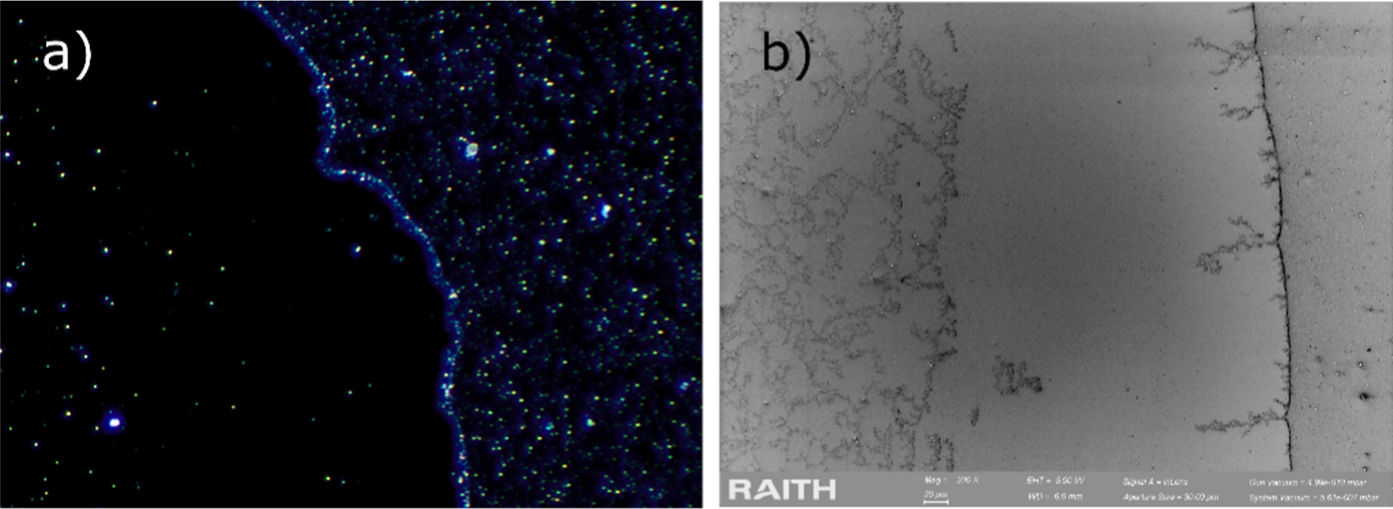
Two examples of deviations from circularity in the perimeter of the droplet imprint in the MWNT material deposit. (a) Optical image, 1 mm size. (b) SEM image, 0.1 mm size.

### 3.6 MWNT Chain Alignment at the End of the CCR Mode

The MWNT chains emerge and align shortly prior to the droplet withdrawal, which implies a direct correlation with the droplet evolution. One factor is the liquid flow (see Section Sd) that could contribute to the MWNT chain behavior. However, the good stability of the MWNT chains makes it plain that the viscous stress from chain movement is limited. Moreover, an inspection of the SEM images that show the connection of the MWNT chains to the perimeter line, as in Figure 3c, leads to the conclusion that while the chains under these conditions have no strong mechanical attachment to the substrate, a flow-induced force does not dominate the alignment of the chains since the same force should drive the chains inward on the droplet surface.

The significant increase in the local curvature of the droplet at the perimeter, as shown in Section 3.5 and prior to the withdrawal stage, is simultaneous with MWNT chain alignment. One effect of the deviatoric curvature Δ*c* is that for an anisotropic particle, the orientational capillary energy of the particle is to leading order of the form^12^

6 ΔE=−CγΔccos⁡2α

where γ is the surface tension and α is the angle between the quadrupolar rise axis on the particle and the major axis of curvature of the droplet surface. The coefficient *C* is positive and depends on the geometry of the particle. In our experiments, the anisotropic particles are MWNTs or rather MWNT/ACP complexes such that the MWNT mainly defines one axis of anisotropy. Thus, according to eq 6, nonzero Δ*c* favors alignment of MWNT chains in the direction perpendicular to the contact line, in agreement with the observed alignment. Furthermore, the nonspherical droplet shape drives particles toward the larger curvature close to the perimeter of the droplet.^12,13^

### 3.7 MWNT Chain Formation

The strong tendency of the MWNTs or MWNT/ACP complexes for end-to-end ordering is obvious in the observed chain and network formations, which are specifically enabled at the air–water interface. This observation has precedents,^12,14,15^ where capillary forces on anisotropic particles at interfaces direct the ordering. Those experiments were performed with ellipsoidal or rodlike particles and are in a larger size category, typically 10 μm in length and a few μm in width. Moreover, optical microscopy could follow individual particles that we cannot do. Nevertheless, the precedents should be comparable to those in our case, as we argue below. Moreover, in our system, the end-to-end and T-connected MWNTs often touch each other via ACPs (cf. theoretical results discussed above). Based on these considerations, we are led to a picture where the MWNT chains are created via capillary interaction together with the ACPs to make structures energetically robust enough for redeposition from the air–water interface to the substrate.

On the theoretical side, the capillary interaction is proportional to the square of the interface deformation amplitude at a particle.^16^ At long distances, the capillary interactions direct the motion of the MWNT/ACP complexes favoring end-to-end ordering of the complexes. At very short distances, the van der Waals interaction^9^ and the hydrophobic interaction^11^ as such would favor side-to-side configurations, but the MWNT/ACP complexes are not driven toward such configurations. At present, we cannot estimate the strength of the capillary interactions theoretically since we cannot measure the related capillary amplitudes at the nanoscale. While at first sight, the diameter of around 10 nm of an MWNT might appear not to be enough to lead to significant deformation and large capillary energy as compared to *kT*, the diameter alone does not necessarily dictate the upper limit for it. First, their length is around 1 μm, increasing the potential for a sizable capillary interaction. Second, most MWNTs are better described as MWNT/ACP complexes, and the large size of the ACPs (up to 1 μm) increases the capillary amplitude. The ACPs are irregularly spherical objects but are constrained by very stiff MWNTs to line up so that they can fit into an effectively one-dimensional picture favoring end-to-end ordering.

### 3.8 Comparison with the Coffee-Ring Effect

The complete process of droplet deposition and drying, as observed in this work, has some similarities with the coffee ring (or coffee stain) phenomenon, mentioned already in the Introduction section. The effect arises from a droplet of complex liquid that contains a nonvolatile solute or, more often, colloidal particles, and therefore, the crucial difference in our system is that the MWNT material is strictly confined to the interfaces.

In the coffee ring effect, the evaporation causes internal flow in the droplet that transfers the colloidal particles to its perimeter, where they assemble into a ring shape that follows the contact line. This accumulation enhances the pinning of the contact line to a steady position. Some significant distinct features have been found to occur when the colloidal particles are anisotropic. In many cases, they organize themselves in the ring deposit with the long axis parallel to the contact line. In a few works, the coffee ring phenomenon has been studied in cases where the anisotropic particles are functionalized carbon nanotubes, where the functionalization renders the tubes dispersible.^17–23^ However, the key topic in this article, the MWNT chains and their capillary force-driven dynamical response to the droplet shape evolution, lacks a counterpart among these works since interfacial phenomena have a much smaller role there.

## 4 Conclusions

We have used pristine MWNTs to demonstrate that they can assemble at the air–water interface of sessile droplets into distinctly one-dimensional chain structures that are markedly sensitive to the changes of the droplet shape upon evaporation. The MWNT material consisted of very hydrophobic MWNTs and equally hydrophobic ACPs, which at the air–water interface are conjoined with the MWNTs. We modeled the van der Waals interactions of the MWNT material on the silicon substrate and at the air–water interface to theoretically estimate the energy related to the transfer to the droplet surface and the concomitant appearance of MWNT/ACP complexes. We found that these estimates are in agreement with the experimental results.

The MWNT/ACP complexes were organized further into chain structures due to the capillary interaction between the MWNTs. These MWNT chains, in turn, align radially at the droplet perimeter region, which exhibits a changing profile with a gradient in the surface curvature. The MWNT chains respond to these surface features via capillary interaction and thus act as a dynamic indicator of the droplet evolution. The process was demonstrated for a strongly pinned droplet following the CCR model of evaporation.

In the typical experiment of this work, only a few MWNT chains at a single location were monitored, but the system with MWNTs on the droplet surface has the potential to follow the contact line behavior more thoroughly. For example, it is likely that the MWNT chains could reveal temporal differences in contact angle development in unprecedented detail at different locations and thus enable more comprehensive studies on the dynamics of evaporating droplets.

## Supplementary Material



## Notes

Materials preparation, experimental setup, notes on the aggregations of the uplifted MWNT material, observations of flow patterns in the droplet, imaging of MWNT chain structures, numerical modeling: van der Waals forces using the Lifshitz theory, numerical modeling: droplet shape with gravity, and numerical modeling: droplet shape at pinning (PDF)
