# Supplementary material for: The Water Droplet Contact Line Probed with Multiwalled Carbon Nanotubes at the Air–Water Interface
Source: Langmuir. 2025 Oct 4;41(40):27263–71. doi: 10.1021/acs.langmuir.5c03184 (PMC12604613; doi:10.1021/acs.langmuir.5c03184)
Supplement: Supplementary file 1 [file la5c03184_si_001.pdf]

SUPPORTING INFORMATION

The water droplet contact line probed with multiwalled carbon nanotubes at the air-water interface

Esa Hyyryläinen, Juha Merikoski and Markus Ahlskog*

Department of Physics and Nanoscience Center, University of Jyväskylä, FI-40014, Finland

Contents

(a) Materials preparation
(b) Experimental setup
(c) Notes on the aggregations of the uplifted MWNT material
(d) Observations of flow patterns in the droplet
(e) Imaging of MWNT chain structure structures
(f) Numerical modelling: van der Waals forces using the Lifshitz theory
(g) Numerical modelling: droplet shape with gravity
(h) Numerical modelling: droplet shape at pinning

(a) Materials preparation

Commercially produced arc-discharge grown MWNT powder, either by Sigma-Aldrich or MER Corp. was used to make a dispersion. The Sigma-Aldrich material contains 20-30 % of MWNTs, with diameter and length ranges of 7-12 nm and 0.5-10 μm respectively, the rest being graphitic impurities, as per to the manufacturer. The MER material has similar characteristics. Our own experience from AFM data gives diameter and length ranges of 5-15 nm and 0.5-3 μm, respectively. A measured volume of 1,2-dichloroethane (DCE, anhydrous laboratory grade, Sigma-Aldrich) was added to weighted MWNT powder, to make a dispersion with concentration of 0.5 mg/ml. This was sonicated with a Hielscher UP400S sonicator, for at least twenty minutes, with sonicating cycle 0.5 s and amplitude 60 %. Diluted dispersions of concentration 0.1 mg/ml were made by adding DCE.

The substrate chips of 8x8 mm size were cut from conventional silicon wafer, either with diamond pen or dicing saw, while the wafer was protected by spin coated PMMA-resist. The chips were cleaned in a beaker containing acetone and sonicated for 3 min in a FinnSonic bath sonicator, followed by rubbing with a cleaning stick in acetone and again in isopropyl alcohol, and drying in a nitrogen stream. The chips were made hydrophilic by reactive ion etching (RIE), in an Oxford Instruments PlasLabs80Plus, in oxygen plasma for two minutes with 200 W forward power, 40 mTorr pressure and 50 sccm gas flow.

Deposition of the MWNT dispersion via spin coating was done immediately after this. The dispersion had been sonicated for at least twenty minutes to prevent aggregation. Droplets of the dispersion were pipetted on the chip in a spin coater at 3000 rpm. The uniformity of deposition was checked with optical microscope. We investigated separately these depositions with different concentrations of the MWNT dispersion and different number (1-8) of spin coating runs. The average density of MWNTs, as counted on 10 μm^2 areas, is shown in the graph of Fig. S1. At all densities, the deposits stayed two dimensional, that is, with no pile up of the material. We concluded that the choice of dispersions of concentration 0.1 mg/ml gave reliable MWNT deposits with which to do the main experiments. As is shown in section c below, the low-density deposits turned out to be of prime interest in this work, and thus most of the samples we report have been made with 2 rounds of spin coating of MWNT dispersion.

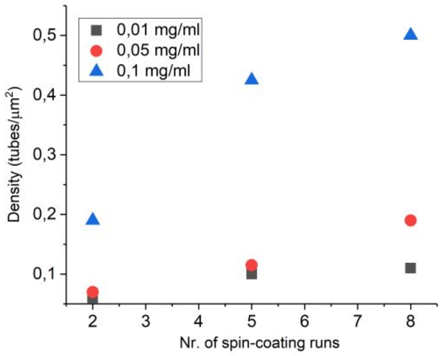

Figure S1. Test data on the density of MWNTs, when deposited from three different dispersions of different concentration, and with a different number of spin coating runs.

(b) Experimental setup

The experiments were done either in a Plas-Labs 830-ABC/EXP glove box or in a small homemade humidity chamber (a cylinder of dimensions 8 cm x 15 cm). The humidity of the former was controlled with Plas-Labs Humidity control box MODEL 800-HCB/EXP, and the latter with saturated salt-solution in a beaker inside the chamber. The humidity in the glove box was measured with its internal humidity probe or a Vaisala HMT333 humidity probe. The humidity in the homemade chamber was measured with an Ahlborn FH0D 46-C humidity sensor, connected to an ALMEMO 2690 datalogger. For modest temperature control, samples could be placed on an Adaptive DT-AR-014-12 thermoelectric Peltier cooler module assembly, with an Adaptive Junior ADJ-48-450-UR temperature controller. A K-type thermocouple was attached to the sample mount with a metallic clamp and GE Varnish.

A droplet of Millipure ultrapure water, was dropped on the Si chip from a pipette. The evolution, that is drying, of the droplet was followed from above via video from an Infinity, model InfiniMax long-range microscope, with MX-1 or MX-5 objectives, Intralux 4000-1 light source, and Olympus SC100 camera. The homemade humidity chamber had optical access to outside inspection via the long-range microscope. The exposure time was usually quite long, one second. After the experiments, optical micrographs of the imprint on the MWNT deposit were taken, using dark-field mode of an Olympus BX53M optical microscope. Scanning electron microscope (SEM) images were taken with Raith e-LiNE Scanning Electron Microscope.

(c) Notes on the aggregations of the uplifted MWNT material

In Figure S2 below is recapitulated (Fig. 2 of the main text) the different steps of the experiment, from droplet placement to its drying, where the main phases are the expansion, standing, and withdrawal phases. The scheme depicts the behavior of the bulk part of the displaced MWNT material, uplifted onto the droplet surface. For clarity, the MWNT chains are not shown here. We present here aspects on how this bulk part behaves, which is not discussed in the main text.

Nearly all reported experiments were done with a 3 μL droplet. The graph in Fig. S2 presents data on how the dimensions of the annulus depend on the droplet volume. While the data of the graph is not surprising, it demonstrates the rather good repeatability of the experiments.

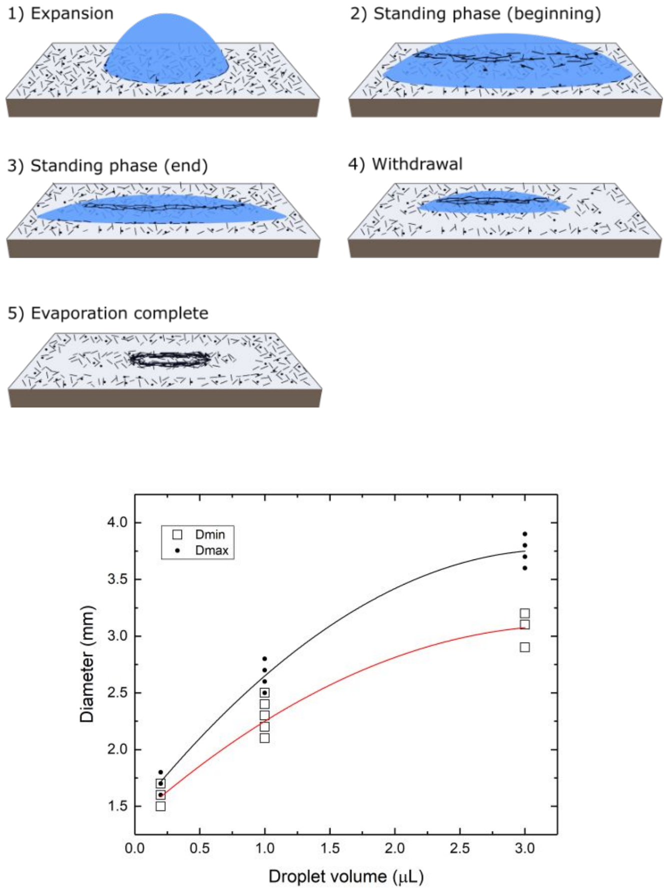

Figure S2. Schematic illustration of the different phases of the experiment with water droplet on a MWNT deposition (black sticks). The different steps are explained in the main text. The graph presents data for the outer (Dmax) and inner (Dmin) diameter of the annulus in the imprint of the MWNT material deposit, as a function of droplet volume.

High vs. low density deposit of MWNT material

During the standing phase most of the MWNT material that is lifted on the droplet surface moves away from its perimeter to dense raft formations towards the center of the droplet (phases 2-3 in Fig. S2). In this work, at the focus is the residual amount of MWNT material that lingers at the perimeter of the droplet surface and forms narrow aggregates and then chain structures (Fig. 4 in the main text). We show next that these aggregates appear reliably only with low densities of the original MWNT material deposit. Figure S3 shows video still images via optical microscopy at the droplet perimeter during the standing phase, from a little wider area than that of Fig. 4 in the main text. The two figure sets are from samples with a high density MWNT deposit (upper set) and with a low density one (lower set). The difference between them is obvious.

In the high-density sample, the uplifted MWNT material forms a dense floating raft of MWNT network, that early in the standing phase extends all the way to the contact line. At a later stage, there is a relatively "clean cut" to the outer perimeter of the annulus, leaving very little residual MWNT aggregates at this boundary. The reason is seemingly that the uplifted MWNT material is dense enough to immediately form some network, which drags away all the uplifted material. Another consequence of the high density is its optical impenetrability. As the raft moves inwards, first the annulus and then, in the last two images, the edge of the initial MWNT deposit is uncovered.

In the low-density sample, the uplifted MWNT material forms mobile MWNT material fragments on the water surface, that in the early stage are not dense enough to aggregate into networks, and thus the initial MWNT deposit inside the annulus region is visible all the time. Moreover, while these

MWNT fragments predominantly slowly drift inwards, occasionally they are absorbed by the contact line. Hence, the key difference between the high- and low-density samples is that with the latter, the perimeter is populated with small MWNT aggregates, that stem from the small share that are attracted by the contact line rather than drifting inwards. Since the MWNT chain structures originate in these perimeter aggregates, our focus on low density samples is thus explained.

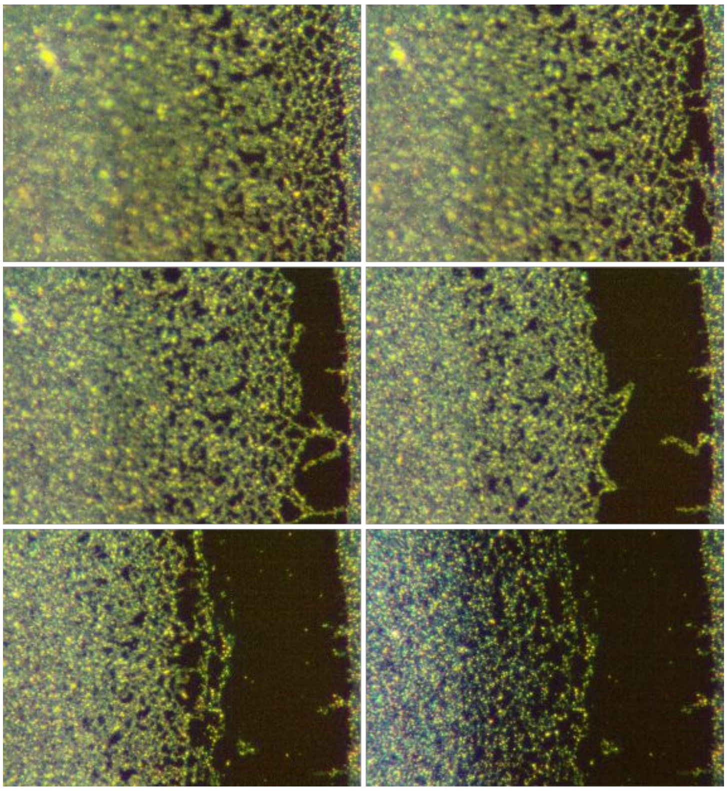

HIGH DENSITY MWNT DEPOSIT

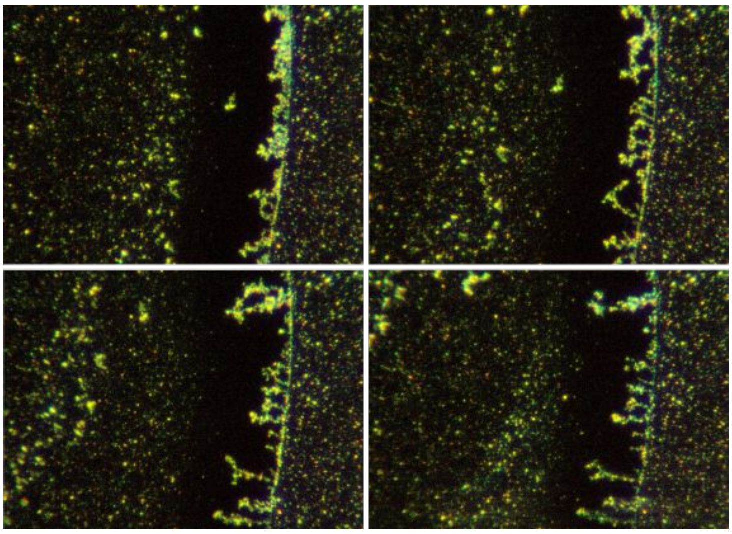

LOW DENSITY MWNT DEPOSIT

Figure S3. Two sets of video-still images via optical microscopy, towards the end of the standing phase, and close to the droplet perimeter. The upper set is from a sample with high density MWNT deposit and the lower set with low density. The droplet is on the left side of each image. The contact line goes along the bright vertical traced line, slightly tilted to the right in the lower set, and very close to the right edge in the upper set. In the low density sample the original deposit is visible all the time below the uplifted MWNT material, while in the high-density sample it becomes visible only in the last two images.

The reposited MWNT material

Subfigure 5 in Fig. S2 includes a depiction of the redeposited bulk part of the uplifted MWNT material after the droplet has dried. The redeposition can be seen inside the annulus as the bright colored, often ring-shaped overlay, like in Figs. 2 and 3 in the main text. Other different outcomes are demonstrated in Fig. S4.

The redeposition process is affected by different parameters (droplet volume, humidity level etc.). Its formation is primarily a question of phenomena such as surface diffusion and aggregation. The experiments were carried out at different RH-values of 10 and 60 %, and the evaporation rate was of course dependent on this. This again determined the time available for aggregation and diffusion of the MWNT material towards the droplet center. Since the droplet expansion phase (1) in all cases removes nearly all the MWNT material it encounters, the redeposited amount depends on the original deposition density. Therefore, the higher the density of the initial deposition, the brighter and wider is the overlay ring.

The first two subfigures of Fig. S4 show two cases with low deposition density. In a), the redeposit has concentrated in a smaller and asymmetrically placed part, while in b), the uplifted MWNT material has not moved into the center part until the droplet has dried and redeposition has occurred. The difference can be explained as a competition between a tendency of the uplifted MWNT material to diffuse towards the center and aggregate, and on the other hand, the evaporation which will ultimately terminate this process upon drying. Therefore, in a), the aggregation process was roughly completed before redeposition, while in the second one, the redeposition has “frozen” the same process at an early phase. The asymmetric position in a) stems simply from the shift of the droplet in the very last stage of evaporation. The latter two subfigures (c,d) come from two high density samples, that repeat the same effects, just with much larger amount of redeposited MWNT material.

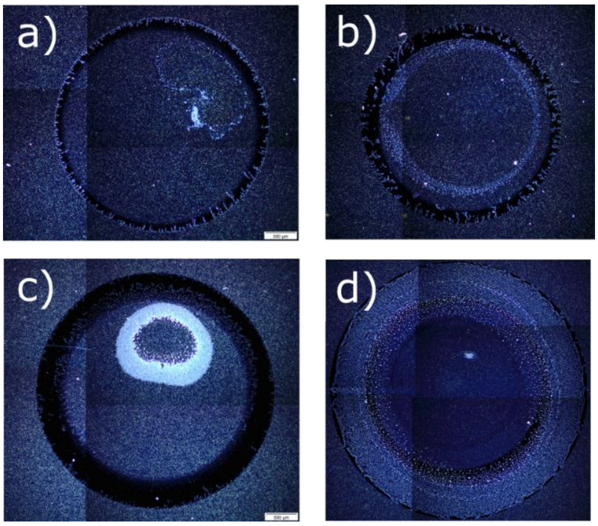

Figure S4. Stitched optical microscopy images with redeposited MWNT material, which is the brighter part in each image. a & b) low density c,d) high density of original MWNT deposit.

(d) Observations of flow patterns in the droplet

Figure S5 shows video still images from the late part of the standing phase, when MWNT chains have straightened out. The images are within 12 sec. The arrow points to an individual particle that moves radially towards the perimeter. From the last two images one can deduce that the particle moves beneath the MWNT chain and therefore demonstrates outward directed flow below the droplet surface.

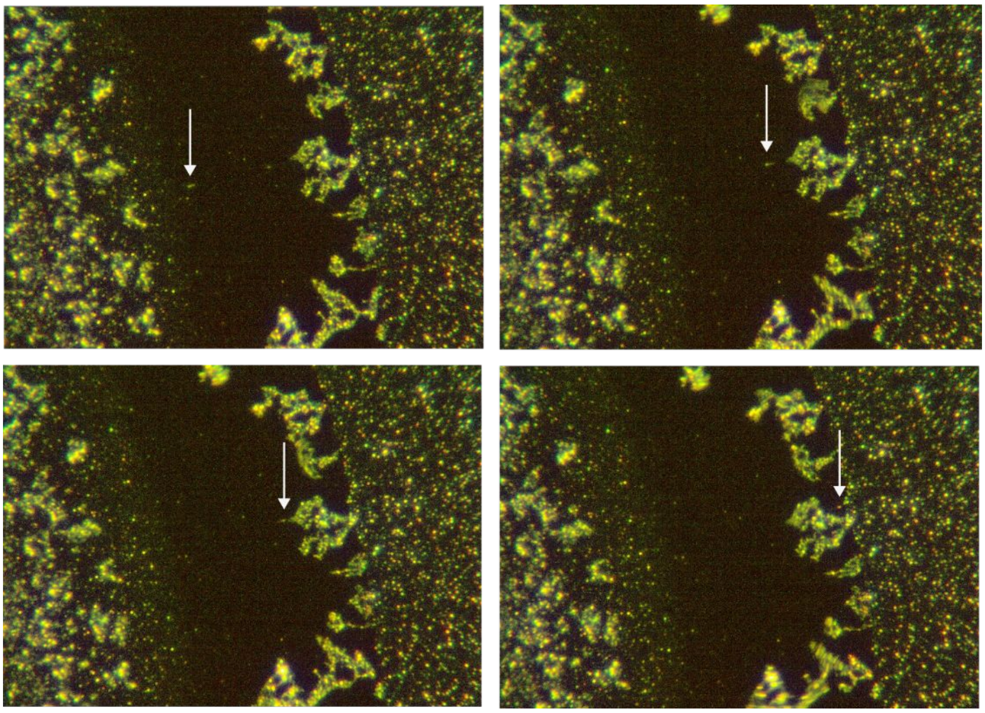

Figure S5. Video still images from late part of the standing phase, see text for explanation.

(e) Imaging of MWNT chain structures

The SEM is needed to obtain more accurate image data of the MWNT chain formations of this work. Figure S6 gives additional image data, to the results presented in section 3.2, on how all ACP particles attach to the MWNTs on the droplet surface, forming MWNT/ACP complexes. The figure demonstrates how the original MWNT material, in (a), undergoes drastic changes in its internal state of aggregation, as it is first uplifted to the air-water interface of the droplet, and then arrange themselves into chain or net-work structures, and are finally redeposited to the Si surface, as shown in (b).

In a few cases we have both optical and SEM image data of the same chain structure, as is demonstrated below in Figure S7. In the sequence of images, first is a wide but high-resolution optical view of redeposited MWNT chain structures, and next to it an enlargement of a much smaller area on a single chain structure. Lastly is a SEM image of the upper part of the same area. A careful comparison between the images demonstrates the dependence of the optical visibility on the ACP coverage of the MWNTs.

Another observation that can be made in the SEM image, are the few separate clean MWNTs that roughly are directed in a northwest – southeast direction. These are tubes that were left on the substrate as the advancing contact line moved over them, in the purification process that was the topic of our previous work. Hence these tubes were never uplifted onto the water surface.

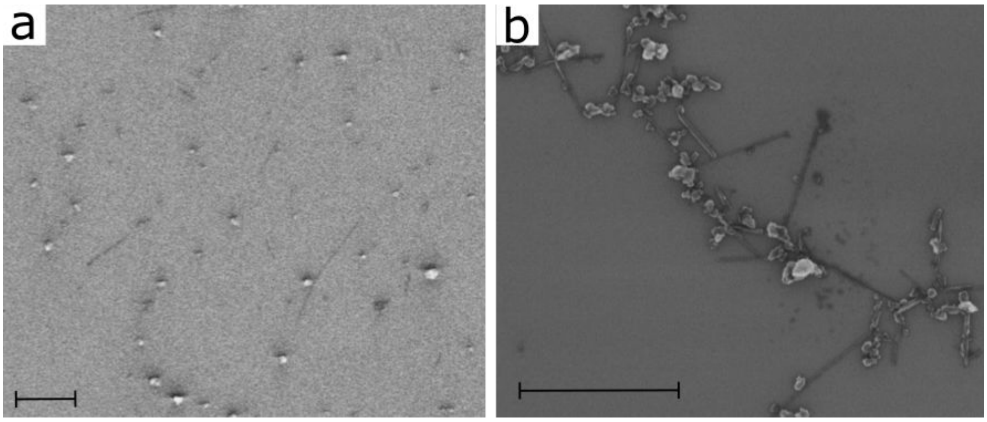

Figure S6. SEM image data, that demonstrates the changes in the internal state of aggregation of the MWNT material, in typical samples before (a) and after (b) exposure to the water droplet. The images are not from the same location, but the picture is very uniformly similar everywhere. Scale bar: 1 μm.

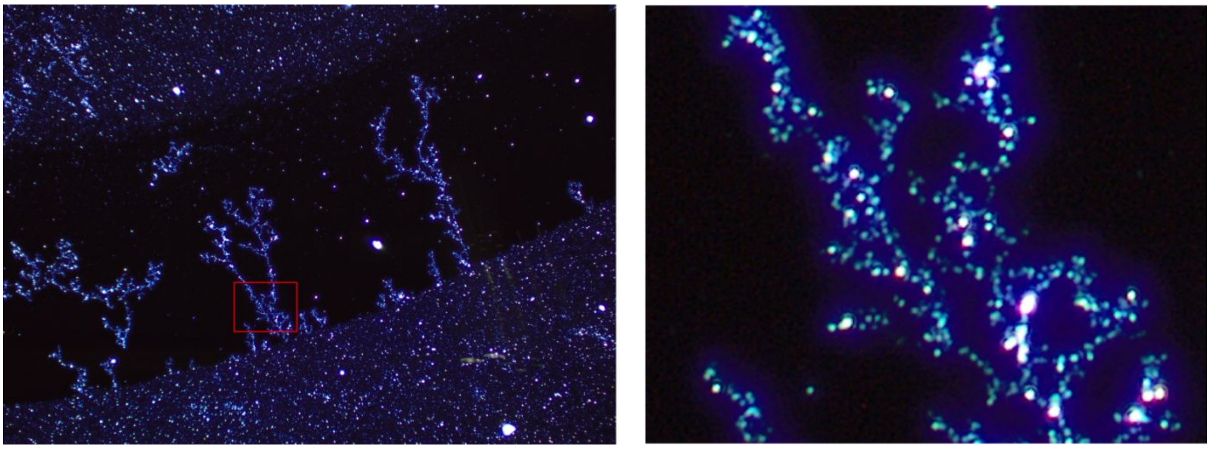

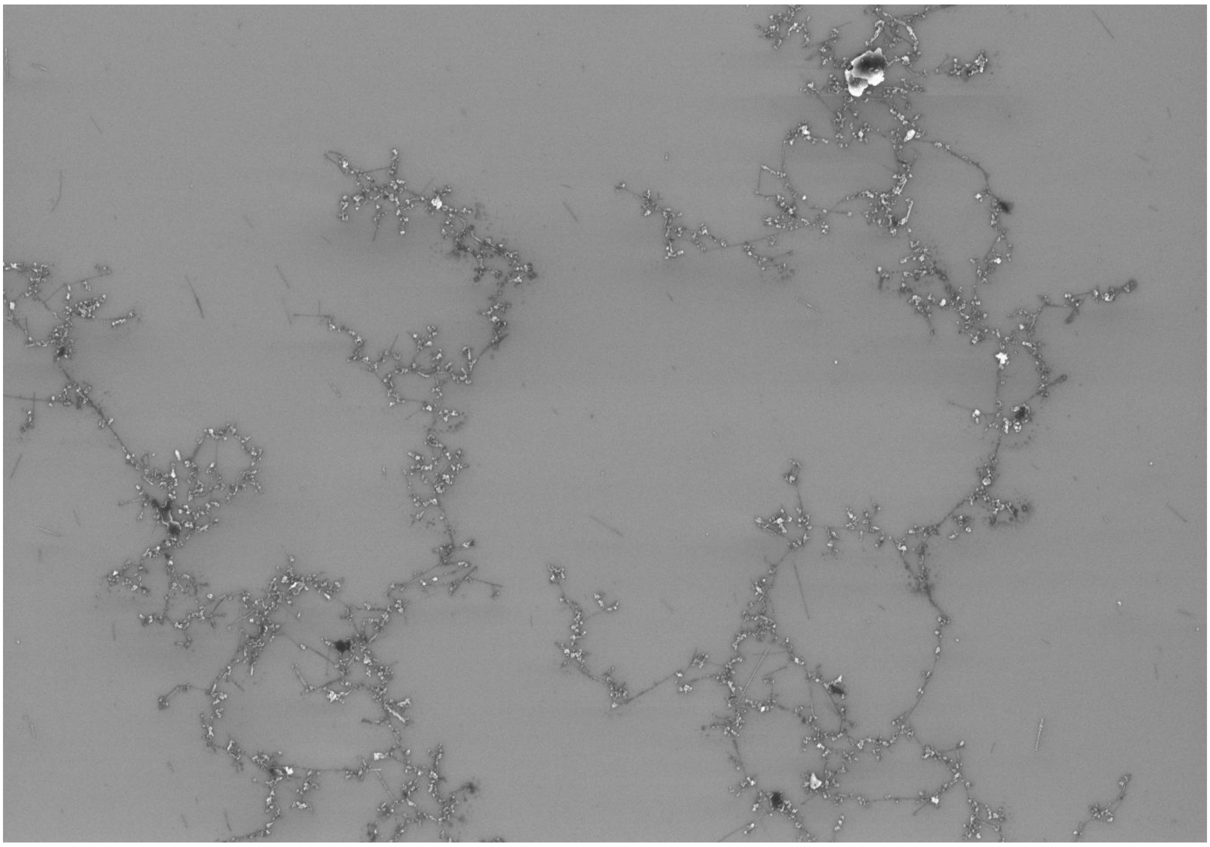

Figure S7. Above: Optical micrograph (left) and enlargement (right) of the area marked with red rectangle. Below: SEM image of the upper part of the red marked area in the optical micrograph.

(f) Numerical modelling: van der Waals forces using the Lifshitz theory with retardation

In computations we use the full Lifshitz formulation with retardation and for curved interfaces with the Derjaguin approximation,[S1] the exception being the simple nonretarded Hamaker approximation used in the case S-C in Table 1 below. The required formulas are given in Eqs. (1-4) of the main text.

We first test our methods using the experimental data by Kocherlakota et al.[S2] as a reference. In Fig. S8 we compare their experimental data for the Hamaker coefficient A with our numerical results based on equation P.2.c.1 in Ref. S1, using the parametrizations of the dielectric functions given for graphite and SiO_2 in the Supplement of Ref. S3. In the experiment of Ref. S2, the Hamaker coefficient A for a stack of graphitic layers (number of layers 1,3,4,6,13) on SiO_2 was measured and in Fig. S8 the experimental data is indicated by the black color. In Fig. S8, we show also our theoretical results for two parametrizations for graphite, calculated from the dielectric function in the directions perpendicular and parallel to the C-axis (S3), indicated by red and blue color, respectively. In our model, the distance between adjacent layers is that of bulk graphite ($c/2$ in standard notation) and the distance of the closest graphite layer from the SiO_2 substrate has been adjusted such that the 'border' between the first and second graphite layer corresponds to the measured height of the (single layer) graphene in the experiment. Thus, there are no free distance parameters in our model here. Below, we shall use the model for the case 'perpendicular', while the case 'parallel' is shown here for comparison, since we discuss later also results for more complicated objects (the difference between our results for the two cases is not very large). We note also that, evidently, a model based on the bulk properties of graphite cannot be an accurate model for a small number of layers (see also the discussion of this in Ref. S2), but our theoretical results are still quite good and good enough for our purposes.

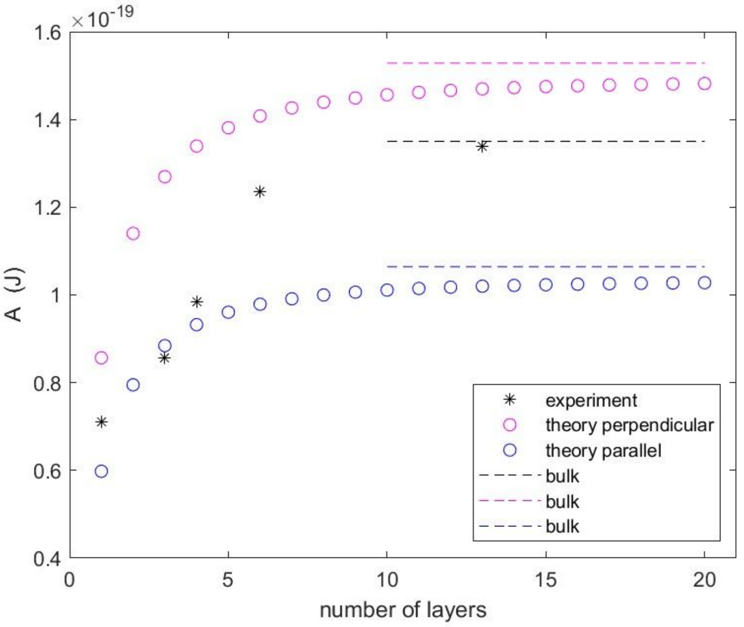

Figure S8. Comparison of experimental data from Ref. S2 and our numerical results for graphite on SiO_2. In the experiments the number of layers 1 means graphene (a single layer) and for larger number of layers the system is HOPG. The Hamaker coefficient A refers to the standard theory such that the free energy of the van der Waals attraction between the graphite layer and the substrate is proportional to A. The experimental data is denoted by black color. Our two theoretical data sets are denoted by red and blue color, the red color corresponding to the parametrization relevant to the experimental situation. Bulk limits for experimental and theoretical results are taken from Ref. S2 and from our computations, respectively.

From the point of view of our further computations discussed later, the comparison with experimental results in Fig. S8 gives some confidence in the order-of-magnitude estimates we need for the present work. Also, thin layers are of interest from the point of view of the 'layer' structure of MWNTs. In particular, and according to Fig. S8, the deviation from bulk free energy is small after about five layers, which is easy to understand from the point of view of the form of the theory at small distances.[S1] The few outmost 'layers' or 'walls' of an MWNT give the main contribution to the interaction energy at short distances. For ACPs, the situation is more complex as they contain more and less ordered grains of graphite so that their response is a varying mixture of responses in different directions with respect to the symmetries of the bulk material.

As discussed above, we use the dielectric function for the 'perpendicular' direction in our theoretical estimates for the free energy of interaction for various configurations to get order-of-magnitude estimates (using 'parallel' would reduce the free energy somewhat in all cases considered, see Fig. S8). We use the parametrizations given in the Supplement of Ref. S3 for the functions $\varepsilon(i\xi)$ for the relevant materials needed in computations along the lines of Ref. S1.
For the case of two semi-infinite media with flat interfaces (denoted here by F-F in the bulk case) or a thin layer (L) on a flat (F) surface of bulk matter (the case L-F), the result of the Lifshitz theory is mathematically exact.[S1] For other cases, with cylindrical (C) or spherical (S) symmetries involved, we rely on the Derjaguin transformation leading to an approximation that is good at short distance between the objects of interest.[S1] In our practical application of the theory, C refers to MWNTs and S to graphitic impurities or ACPs.
In Table S1 we summarize the main results of our short-range ($\ell \ll R_j$) calculations for the van der Waals energies of relevant geometric configurations obtained by using the formulas in ref S1 as given in section Materials and Methods/Theory of the main text. The length of a cylinder is taken to be L_C =

μm, a typical length of MWNTs. For the cylinder radius we use the value R_C= 5 nm and for the sphere radius R_S = 20 nm, since these are typical radiuses of MWNTs and ACPs in our experiments. Thus $L_C \gg R_C$ and the effect of the ends of the cylinder on G/L_C is negligible. The distance between various objects in each case is denoted by ℓ. The distances (minimum distances) we use below for distances between nanocarbon and the substrate and between nanocarbon and water are ℓ_S = 0.6646 nm and ℓ_W = 0.32 nm, where the value of ℓ_S is based on the experimental results discussed above for graphite on SiO_2 from Ref. S2 and ℓ_W based on simulation results for SWNTs immersed in of H_2O (S4), respectively. In Table 1 the abbreviation NC means nanocarbon, in our case particles of cylindrical (C) or spherical (S) symmetry, that is, MWNTs and ACPs.

geom.	materials	ℓ	G (eV)	notes	Equation in (S1)
$\mathrm{F-F}$	H_2O - SiO_2 in air	ℓ_S	0.02207	G per area 1 nm², bulk	P.1.a.1
$\mathrm{F-L/F}$	graphite - SiO_2/Si in air	ℓ_S	0.05957	G per area 1 nm², bulk	P.2.a.1
$\mathrm{F-F}$	graphite - SiO_2 in air	ℓ_S	0.05587	G per area 1 nm², bulk	P.1.a.1
$\mathrm{L-F}$	graphite - SiO_2 in air	ℓ_S	0.05177	G per area 1 nm², 5 layers	P.2.c.1
$\mathrm{L-F}$	graphite - SiO_2 in air	ℓ_S	0.03211	G per area 1 nm², 1 layer	P.2.c.1
$\mathrm{C-F}$	NC - SiO_2 in air	ℓ_S	219.7	valid as $L_C \gg R_C \gg \ell$	C.1.b
$\mathrm{S-F}$	NC - SiO_2 in air	ℓ_S	4.175	valid as $R_S \gg \ell$	S.1.b
$\mathrm{C-F}$	NC - H_2O	ℓ_W	527.3	valid as $L_C \gg R_C \gg \ell$	C.1.b
$\mathrm{S-F}$	NC - H_2O	ℓ_W	7.200	valid as $R_S \gg \ell$	S.1.b
$\mathrm{C-F}$	NC - SiO_2 in water	ℓ_S	37.65	valid as $L_C \gg R_C \gg \ell$	C.1.b
$\mathrm{S-F}$	NC - SiO_2 in water	ℓ_S	0.7152	valid as $R_S \gg \ell$	S.1.b
$\mathrm{C-C}$	NC -NC in air	ℓ_S	338.7	valid as $L_C \gg R_C \gg \ell$	C.1.b
$\mathrm{S-C}$	NC -NC in air	ℓ_S	19.92	valid as $L_C \gg R_C \gg \ell$	C.8.c Hamaker
$\mathrm{S-S}$	NC -NC in air	ℓ_S	4.377	valid as $R_S \gg \ell$	S.1.b
$\mathrm{C-C}$	NC -NC in water	ℓ_S	1.844	valid as $L_C \gg R_C \gg \ell$	C.1.b
$\mathrm{S-C}$	NC -NC in water	ℓ_S	10.85	valid as $L_C \gg R_C \gg \ell$	C.8.c Hamaker
$\mathrm{S-S}$	NC -NC in water	ℓ_S	2.432	valid as $R_S \gg \ell$	S.1.b

Table S1. Numerical results for the free energy G of the van der Waals attraction with retardation at given distance ℓ for objects of interest for various geometries and material combinations, with theory valid at short distances $\ell \ll R_j$. In most cases the medium between the objects is vacuum, on the tree last rows it is water. Abbreviation F means a flat surface of bulk material, L means a flat thin layer, and C is an object with cylindrical symmetry and S an object with spherical symmetry. NC means nanocarbon of cylindrical or spherical symmetry. The condition $L_C \gg R_C$ means that the effects of the ends of the nanotubes are negligible and the conditions $R_C \gg \ell$ and $R_S \gg \ell$ are required by the Derjaguin transform.[S1] The values of the parameters are L_C = 1 μm, R_C= 5 nm, R_S = 20 nm, ℓ_S = 0.6646 nm and ℓ_W = 0.32 nm.

We now discuss the applicability of the results above to our experimental system. We have chosen to use parametrization for SiO_2 as the surface of the substrate is SiO_2 (the native oxide layer of thickness 2 nm), while the bulk of the substrate is Si. On the second and third row of the table we compare results for flat interfaces: the difference in G for interaction of graphite between SiO_2/Si and SiO_2 is only 6.6% (this difference depends on ℓ but we use only one value $\ell = \ell_S$ in the cases relevant here). If the surface of graphite is curved, the difference is somewhat larger, but that comparison requires an approximation. We shall continue using the results for SiO_2 since we are interested in the order of magnitude of G in various geometries only.
MWNTs have a loose 'layer' (wall) structure and for a small distance ℓ the dominant contribution to G comes from closest parts of the objects. In our practical application G is lower than the value for a cylinder in Table S1 (c.f. Fig. S8 also). For ACPs we note that the structure of an ACP is very complex, consisting of more and less ordered grains of graphite and disordered material also, so the dielectric

response is mixed (c.f. results ‘perpendicular’ and ‘parallel’ in Fig. S8). Therefore, the energies in our practical application are smaller than the values in Table 1 obtained for homogeneous objects.

We shall take these considerations into account in our final order-of-magnitude estimates denoted by red colour below:

0. For the flat geometries L-F and F-F with graphite and SiO_2 the interaction free energies G per area of 1 nm^2 vary between 0.032 eV (one layer) and 0.056 eV (infinite number of layers). The case of H_2O on SiO_2 is also of some interest, with the corresponding value 0.022 eV.
1. Using a cylinder as a model for an MWNT, its interaction energy on the SiO_2 substrate (C-F) would be G = 220 eV per length of 1 μm. Because of the loose ‘layer’ structure of the MWNT, we make for it the order of magnitude estimate $\sim$100 eV (c.f. the difference between one layer and bulk in the case L-F). For a cylinder of length 1 μm on the surface of water we get G = 520 eV, about twice that on the substrate. For a typical MWNT of length 1 μm the order of magnitude of the attraction in the two cases is thus $\sim$100 eV on the substrate and $\sim$200 eV on water. For an MWNT inside water on the substrate we get by similar arguments $\sim$20 eV.
2. For a spherical particle on the SiO_2 substrate (S-F) we found G = 4.2 eV. ACPs are amorphous and contain small pieces of bulk graphite with different orientations and, also, completely disordered material, so we expect their interaction with the substrate to be considerably weaker and of the order of $\sim$1 eV. On the water surface we got G = 7.2 eV, somewhat larger than on the substrate, so we use $\sim$2 eV. For an ACP inside water on the substrate G = 0.72 eV so we make the estimate $\sim$0.2 eV. We know however that the overall shape of an ACP is very irregular so the effective area of contact with a flat surface can be considerably larger than that for a spherical particle, so here we in most cases probably underestimate the interaction energies. In any case they are larger than kT and well below those for MWNTs.
3. For a spherical particle attached to a cylindrical particle (S-C) we found G = 20 eV with an ideal sphere and an ideal cylinder as the model. Our order of magnitude estimate of the interaction between much less ideal particles, an MWNT and an ACP, would then be $\sim$2 eV in vacuum and $\sim$1 eV inside water. Here also, the shape of an ACP is irregular, so the effective area of contact is larger than that for a spherical particle, so we underestimate the energies.
4. In addition, we show in the table results for two parallel cylinders (C-C) and two spheres (S-S).

An ideal solid cylinder cannot be an accurate model for an MWNT and we have taken that into account in the estimates of the free energy above. One possible approach would be to use as a model a hollow solid cylindrical tube.[S5] A problem with that approach from our point of view is that the material would still be assumed to be bulk material, while a real MWNT has a complicated internal microscopic structure that is very different for each MWNT and cannot be experimentally characterized in detail for the MWNTs we have in our experiments. It is known e.g. that the outer radius and the number of concentric tubes or ‘layers’, also their relative positions, varies a lot. For small distance ℓ, the outmost ‘layers’ are responsible for a major part of the Hamaker coefficient. In our case, the outer radius of the MWNTs (5 nm) is quite large compared with the distance from the surface (0.66 nm). Also, corrections related to the bending of an optically anisotropic material are thus expected to be small. Another approach, also leading to challenges discussed above, would be to set up an ab initio calculation of the optical properties of MWNTs. For SWNTs this has been achieved to such an extent that there are ab initio results for different radiuses and chiralities of SWNTs,[S6] with considerable dependence of the dielectric function on microscopic details of the SWNTs.

For our conclusions in the main text of the article, the essential inputs are the orders of magnitudes of the free energy of the attraction for relevant geometric configurations and their relative order. The effect of using the retarded formulation instead of the simpler nonretarded short-distance limit (Eq. P.1.a.3 in Ref. S1 for F-F and corresponding Eqs. for other geometries) is relatively small for the

distances ℓ of a few Å considered above, as demonstrated in Fig. S9 below, where the Hamaker coefficient $A(\ell)$ as a function of the distance ℓ of objects is shown for various geometries. As expected, compared with flat interfaces (F-F), the effect of ℓ on $A(\ell)$ is larger and appears earlier as a function of the distance ℓ for geometries with curved interfaces (C-F, C-C, S-F, S-C). The short-distance limit can be computed 'exactly in numerical sense' and corresponds to the zero-distance limit of the curves in the figure. The dependence on distance ℓ is expected to become more relevant in comparing longer-range van der Waals interactions with capillary interactions (see the main text of the article), but this is left to future work.

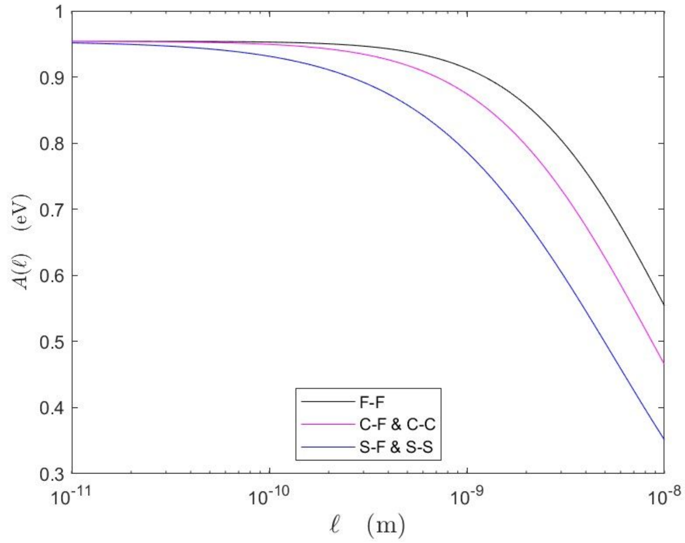

Figure S9. The effect of retardation on the Hamaker coefficient $A(\ell)$ as a function of distance ℓ for graphite on silicon oxide in various geometries. The nonretarded limit is $\ell \to 0$. With particle size relevant to our work, the value $\ell_{max} \sim 10^{-8}$ m is the validity limit of the Derjaguin transform.[S1] The difference in energy G between the cases C-F and C-C (similarly between S-F and S-S) appears only in distance-independent parameters z_k and g_k in G, see Eq. (1) in the main text.

For future reference we list in Table S2 the values of the Hamaker coefficient without retardation, A, and with retardation, $A(\ell)$, for the relevant material combinations and geometries. Depending on geometry the maximum difference between non-retarded and retarded formulations is around 13 %. We prefer to use $A(\ell)$ for better accuracy, with retardation and the Derjaguin transform, which can be expected to be valid for $\ell \ll R_i$, where R_i is the radius of curvature of the object of interest. For objects in our study the validity limit is $\ell \sim 10^{-8}$ m. This means that, with the retarded formulation, interactions between nanocarbon particles could be studied in future for distances higher than those in the present work such that $A(\ell)$ would produce an important part of the ℓ dependence (however, for such a study the capillary interactions are needed which is beyond the scope of the present work as explained in the main text). We note that the values of A favor the contact of C with SiO_2 over H_2O. However, the different distances of the nanocarbon objects from the substrate ℓ_S = 0.6646 nm and from water ℓ_W = 0.32 nm strongly favor the contact with water overriding the difference in A, as computed from the direct ℓ dependence in G for cylindrical and spherical geometry: for an MWCNT ($\ell_W/\ell_S)^{-3/2} \approx 3.0$ and for an ACP $(\ell_W/\ell_S)^{-2} \approx 4.3$.

materials	A	$A(\ell)$ $\mathrm{F-F}$	$A(\ell)$ $\mathrm{C-F/C-C}$	$A(\ell)$ $\mathrm{S-F/S-S}$	max. diff.
C - C in air	1.9696	1.9283	1.8790	1.7453	-11 %
C - SiO_2 in air	0.9540	0.9304	0.9033	0.8323	-13 %
C - H_2O in air	0.7400	0.7342	0.7245	0.6912	-7 %

C - C in water	1.0721	1.0560	1.0341	0.9699	-10 %
C - SiO_2 in water	0.1633	0.1594	0.1548	0.1426	-13 %

Table S2. The Hamaker coefficient without retardation, A, and with retardation, $A(\ell)$, for the relevant material combinations and geometries, given in electron volts (eV). The values of ℓ are as in Table S1 and $A(\ell) \rightarrow A$ for $\ell \rightarrow 0$. The rightmost column gives the maximum difference when going from A to $A(\ell)$.

(g) Numerical modelling: droplet shape with gravity

As explained in the main text of the article, key factors in the energy of particles on a curved interface are the local principal curvatures c_1 and c_2 and the deviatoric curvature $\Delta c = c_1 - c_2$ of the interface, in our experimental system the interface between the liquid and gas phases. Figure S10(a) shows the profile of an ideal droplet (a typical case from experiments) in the presence of gravity as given by the Young-Laplace equation. We solved the equation along the lines presented in Ref. S7, for a single representative experiment. As experimental input to the calculation we used the observed contact angle θ = 0.35, the droplet radius R = 2 mm and the droplet volume V = 2.8 μl. As the solution for the droplet shape from the Young-Laplace equation has only two parameters (the Bond number and the contact angle), we optimized the shape by minimizing the weighed (by experimental accuracy) sum of the squares of relative deviations of θ, R and V from our experimental values. We take the value of the surface tension, γ = 0.076 N/m, corresponding to our experimental conditions from ref S8. For the Bond number $\mathrm{Bo} = \Delta\rho g R_0^2/\gamma$ our optimized solution leads to the value Bo = 4.5, where $\Delta\rho$ is the density difference of the fluid phases, g is the acceleration of gravity and R_0 is the curvature at the apex of the droplet. This value indicates that gravity may be of interest. Fig. S10(b) shows the two principal curvatures c_1 and c_2 and Fig. S10(c) shows the deviatoric curvature $\Delta c = c_1 - c_2$. The droplet gets flattened as compared with the shape of a spherical cap. Due to the gravity-induced changes in the local curvature, MWNT/ACP complexes should be driven towards the contact line and be directed along the direction perpendicular the contact line corresponding to c_1 (discussion in the [illegible]

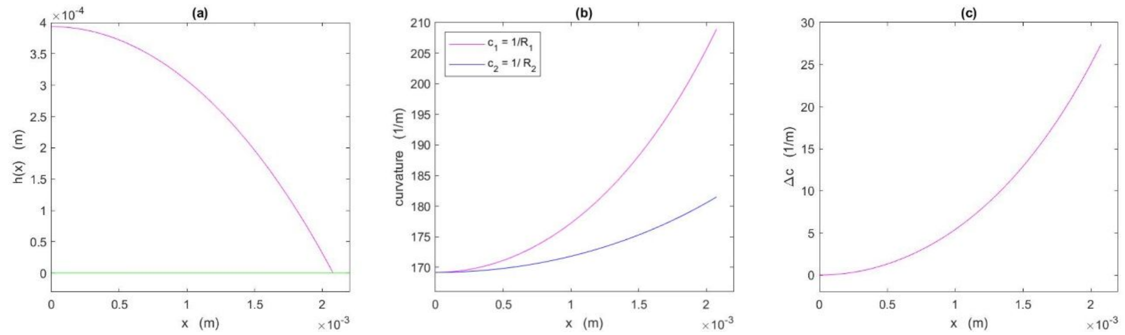

Figure S10. (a) Droplet profile $h = h(x)$ from the Young-Laplace equation as fitted to experimental parameters. The eccentricity of this droplet shape is 0.72. Here x is the horizontal coordinate and the green line indicates the surface of the substrate. (b) The principal curvatures c_1 and c_2. (c) The deviatoric curvature $\Delta c = c_1 - c_2$.

(h) Numerical modelling: droplet shape at pinning

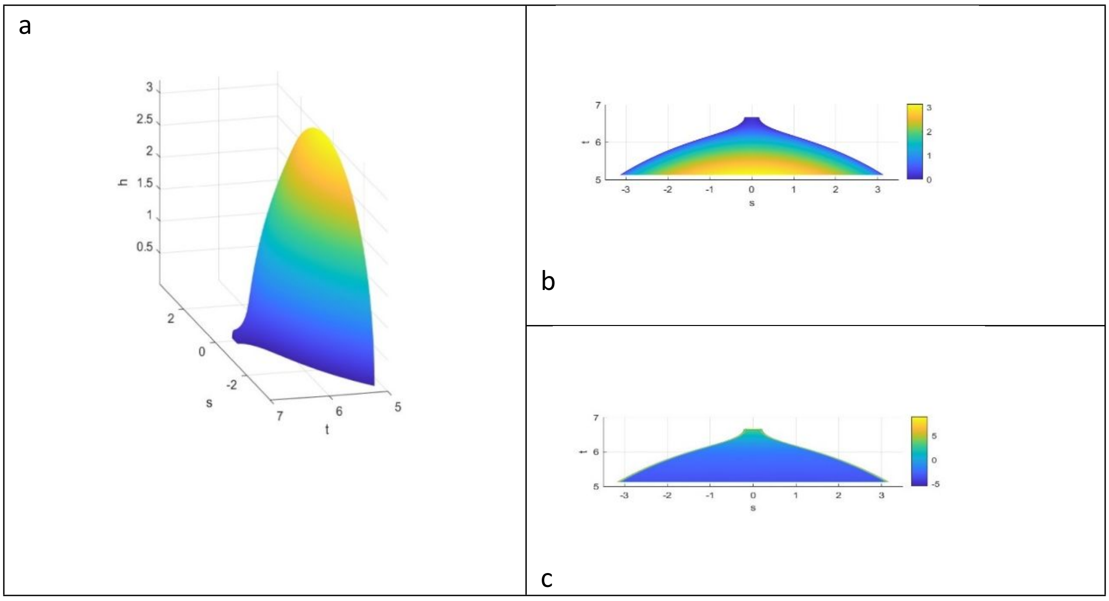

Fig S11. (a) Part of a droplet surface with a locally pinned contact line (the model is described in text). (b) The droplet seen from above. (c) Logarithm of the deviatoric curvature $\log\Delta c$ with $\Delta c = c_1 - c_2$. In (c) a narrow strip of yellow color is seen at the contact line, which is a numerical artefact.

During evaporation with pinning, the shape of the droplet gets increasingly different from that of a spherical cap, which affects the local mean curvature and Δc. In Fig. S11(a) we show a part of a surface of an evaporating droplet, where the contact line is locally pinned (examples of pinning are seen in images of the main text). In Fig. S11(b) the same droplet is seen from above to display the shape of the contact line. In Fig. S11(c) we show the deviatoric curvature demonstrating the local increase in the deviatoric curvature Δc due to the pinning. Note that we show $\log\Delta c$ so the relative differences in Δc are large. The droplet surface shown in Fig. S11(a) was constructed such that its mean curvature $(c_1 + c_2)/2$ is constant as required by Laplace law with constant pressure difference Δp, meaning that the droplet has an equilibrium shape as the contact line stays locally pinned for a while. The shape of the contact line in Fig. S11(a) is quite like that discussed in Section 3.3 of Ref. S9 with the deviation from a circular shape being logarithmic in distance. Note that, compared to a realistic situation, the size of the actual pinning region compared with the droplet size has been exaggerated in Fig. S11(a) to make the overall shape of the pinning region and its deviation from circular shape clearly visible. In the calculation below we use more realistic values of parameters (with smaller pinning region).
Assume now that pinning is caused by an MWNT on the substrate such that the MWNT is oriented in direction perpendicular to the contact line (orientation consistent with experiment). The extra area of the droplet surface due to pinning in the model above, of length L = 1 nm and radius $R =$ 5 nm, can be estimated to be of the order of $\pi RL \approx$ 16 nm². Multiplying this by the surface tension of water (see above), γ = 0.076 J/m², to find the cost of the pinned region in interface free energy, we get 8 eV. According to our calculations (see Table 1 above and discussion) the interaction of typical MWNTs with the substrate or with water surface is of the order of 100 eV, larger for the water surface than the substrate surface. We conclude that MWNTs are expected to be quite immobile and prefer to stay in contact with water at the contact line, so they can be responsible for the pinning of the contact line. Similar conclusion applies to MWNT/ACP complexes.

References for the Supporting Information

(S1) Parsegian, V. A. *Van der Waals Forces* (Cambridge 2006),

https://doi.org/10.1017/CBO9780511614606
(S2) Kocherlakota, L. S.; Krajina, B. A.; and Overney, R. M. Local energetic analysis of the interfacial and surface energies of graphene from the single layer to graphite, J. Chem. Phys. **2015**, *143*, 241105-1-5. *https://doi.org/10.1063/1.4939248*
(S3) Gudarzi, M. M.; Aboutalebi, S. H. Self-consistent dielectric functions of materials: Toward accurate computation of Casimir–van der Waals forces, Sci. Adv. **2021**, *7*, 1-8. *https://doi.org/10.1126/sciadv.abg2272*
(S4) Walther, J. H. ; Jaffe, R.; Halicioglu, T.; Koumoutsakos, P. Carbon Nanotubes in Water: Structural Characteristics and Energetics, J. Phys. Chem. B **2001**, *105*, 9980-9987. *https://pubs.acs.org/doi/10.1021/jp011344u*
(S5) Šiber, A.; Rajter, R. F.; French, R. H.; Ching, W. Y.; Parsegian, V. A.; Podgornik, R. Dispersion interactions between optically anisotropic cylinders at all separations: Retardation effects for insulating and semiconducting single-wall carbon nanotubes, Phys. Rev. B **2009**, *80*, 165414-1-10. *https://doi.org/10.1103/PhysRevB.80.165414*
(S6) Rajter, R. F.; French, R. F.; Ching, W. Y.; Podgornik R.; Parsegian, V. A. Chirality-dependent properties of carbon nanotubes: electronic structure, optical dispersion properties, Hamaker coefficients and van der Waals–London dispersion interactions, RSC Adv*.* **2013**, *3*, 823-842. *https://doi.org/10.1039/C2RA20083J*
(S7) Yang, M. W.; Lin, S. Y. A method for correcting the contact angle from the θ/2 method, Colloids and Surfaces A: Physicochem. Eng. Aspects **2003**, *220*, 199-210. *https://doi.org/10.1016/S0927-7757(03)00064-5*
(S8) Pérez-Díaz, J.; Álvarez-Velenzuela, M.; García-Prada; J. C. The effect of the partial pressure of water vapor on the surface tension of the liquid water–air interface, J. Colloid Interface Sci. **2012**, 381, 180-182. *https://doi.org/10.1016/j.jcis.2012.05.034*
(S9) deGennes, P-G.; Brochard-Wyart, F.; Quere, D. Capillarity and Wetting Phenomena: Drops, Bubbles, Pearls, Waves (Springer 2003).
